# Asymmetric Synthesis of Spirocyclic 2-Benzopyrans for Positron Emission Tomography of σ_1_ Receptors in the Brain

**DOI:** 10.3390/ph7010078

**Published:** 2014-01-22

**Authors:** Katharina Holl, Dirk Schepmann, Steffen Fischer, Friedrich-Alexander Ludwig, Achim Hiller, Cornelius K. Donat, Winnie Deuther-Conrad, Peter Brust, Bernhard Wünsch

**Affiliations:** 1 Institut für Pharmazeutische und Medizinische Chemie der Westfälischen Wilhelms-Universität Münster, Corrensstraße 48, Münster D-48149, Germany; 2 Helmholtz-Zentrum Dresden-Rossendorf, Institut für Radiopharmazeutische Krebsforschung, Forschungsstelle Leipzig, Abteilung Neuroradiopharmaka, Permoserstraße 15, Leipzig D-04318, Germany

**Keywords:** 2-benzopyrans, Sharpless Asymmetric Dihydroxylation, spirocycles, σ affinity, radiochemistry, positron emission tomography, autoradiography, organ distribution

## Abstract

*Sharpless* asymmetric dihydroxylation of styrene derivative **6** afforded chiral triols **(*R*)-7** and **(*S*)-7**, which were cyclized with tosyl chloride in the presence of Bu_2_SnO to provide 2-benzopyrans **(*R*)-4** and **(*S*)-4** with high regioselectivity. The additional hydroxy moiety in the 4-position was exploited for the introduction of various substituents. Williamson ether synthesis and replacement of the Boc protective group with a benzyl moiety led to potent σ_1_ ligands with high σ_1_/σ_2_-selectivity. With exception of the ethoxy derivative **16**, the (*R*)-configured enantiomers represent eutomers with eudismic ratios of up to 29 for the ester **(*R*)-18**. The methyl ether **(*R*)-15** represents the most potent σ_1_ ligand of this series of compounds, with a K_i_ value of 1.2 nM and an eudismic ratio of 7. Tosylate **(*R*)-21** was used as precursor for the radiosynthesis of [^18^F]-**(*R*)**-**20**, which was available by nucleophilic substitution with K[^18^F]F K222 carbonate complex. The radiochemical yield of [^18^F]-**(*R*)-20** was 18%–20%, the radiochemical purity greater than 97% and the specific radioactivity 175–300 GBq/µmol. Although radiometabolites were detected in plasma, urine and liver samples, radiometabolites were not found in brain samples. After 30 min, the uptake of the radiotracer in the brain was 3.4% of injected dose per gram of tissue and could be reduced by coadministration of the σ_1_ antagonist haloperidol. [^18^F]-**(*R*)**-**20** was able to label those regions of the brain, which were reported to have high density of σ_1_ receptors.

## 1. Introduction

The σ receptor was firstly described in 1976 by Martin *et al*. It was named after the ligand SKF-10,047 and initially regarded as opioid receptor subtype [1]. Further research resulted in the classification of σ receptors as a distinct receptor class. In 1990, the existence of at least two σ receptor subtypes was discovered, which were named σ_1_ and σ_2_ receptor [2]. The σ_1_ receptor has been cloned from different species and tissues including guinea pig liver [3], mouse and rat brain and a human placental tumor cell line. The transmembrane protein consists of 223 amino acids [4] with a molecular weight of 25.3 kDa. A homology with another known mammalian protein was not found, but a 30% homology with the yeast enzyme sterol-Δ^8^/Δ^7^-ismerase, encoded by the gene ERG2, was detected [3]. The σ_1_ receptor has not been crystallized so far, but a structural model was published in 2002 [5] and a 3D homology model was established in 2011 [6]. σ_1_ receptors are found in the central nervous system [7], but also in peripheral organs, like liver, kidney [8] and heart [9]. Endogenous ligands have not been clearly identified so far, although some neurosteroids (e.g., progesterone, dehydro-epiandrosterone) and *N,N*-dimethyltryptamine were proposed as endogenous ligands [10,11]. The σ_1_ receptor is supposed to have an influence the permeability of ion channels [12,13] and the activity of neurotransmitter systems [14,15]. In 2007, Hayashi and Su postulated the role of the σ_1_ receptor as a ligand-operated chaperon [16].

Because of the manifold modulatory effects of the σ_1_ receptor, potent and selective σ_1_ receptor ligands represent potential therapeutics mainly for neurological and psychiatric diseases such as Alzheimer’s Disease [17], neuropathic pain [18], schizophrenia [19,20] and Major Depression [15,21,22]. PET tracers, which are able to label selectively σ_1_ receptors, are of high interest not only to gain further insight into the physiological role of the σ_1_ receptor, but also for the diagnosis of diseases in which the σ_1_ receptor is involved.

**Figure 1:**
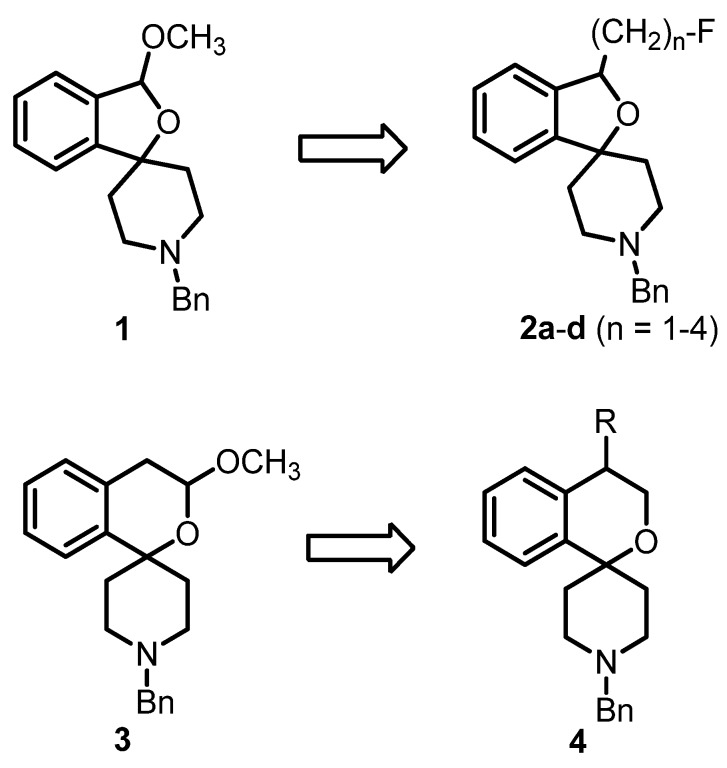
Development of fluorinated PET tracers

A number of PET tracers for the imaging of σ_1_ receptors, labeled with [11C] or [^18^F], have already been developed [23,24,25,26]. Very recently, we have reported on the homologous series of fluorinated spirocyclic piperidines **2a**–**d** (n = 1–4, Figure 1), which were derived from the potent σ_1_ receptor antagonist **1**. The use of the 2-benzofuran-based [^18^F]-labeled spirocyclic σ_1_ receptor ligands [^18^F]**2** was carefully evaluated *in vivo* [27,28,29,30,31]. Moreover, (*R*)- and (*S*)-configured enantiomers of **2a**–**c** (n = 1–3) were prepared and it was shown that the corresponding enantiomers differ considerably in σ_1_ receptor affinity, selectivity over the σ_2_ subtype, rate of biotransformation and number and nature of formed metabolites. Additionally, the accumulation of the enantiomeric fluorinated PET tracers in the central nervous system was considerably different [32,33,34].

The fluoroalkyl substituted 2-benzofurans **2a**–**d** (n = 1–4) were derived from the spirocyclic 2-benzofuran **1**; the potent σ_1_ antagonist **3** represents the lead for the enantiomerically pure 2-benzopyrans **4**. Although the 2-benzopyran **3** (K_i_ = 1.3 nM) represents a very potent σ_1_ receptor antagonist [35], enantiomers of 2-benzopyran based σ_1_ ligands were not yet investigated. Due to their structural similarity to the spirocyclic 3-substituted 2-benzopyrans **3** [36] and 2-benzofurans **1** and **2**, 4-substituted 2-benzopyrans **4** were considered as new type of σ_1_ receptor ligands. Moreover, the 2-benzopyran scaffold was not exploited for the development of a fluorinated PET tracer so far. In this communication we report the first enantioselective synthesis of 4-substituted spirocyclic 2-benzopyrans of type **4**, their affinity towards σ receptors and the generation and biological evaluation of a [^18^F]-labeled PET tracer based on this scaffold.

## 2. Experimental

### 2.1. Synthesis

#### 2.1.1. General

Solvents: THF: distilled from sodium/benzophenone; CH_2_Cl_2_: distilled from calcium hydride. Flash chromatography: silica gel 60 (40–63 µm); parentheses include: diameter of the column (ø), height of the stationary phase (*h*), eluent and fraction size (*V*). Thin layer chromatography: TLC silica gel 60 F_254_ on aluminum sheets. Melting points (mp): uncorrected. Polarimetry: sodium D line (589 nm); length (*l*): 1 dm; temperature +20 °C; unit of specific rotation [deg mL dm^−1^ g^−1^] is omitted; parentheses include: concentration of the sample [mg/mL] and solvent: ^1^H-NMR: 400.3 MHz; ^13^C-NMR: 100.3 MHz; chemical shifts in [ppm] against TMS; in some cases, ^1^H and ^13^C-NMR spectroscopy were supported by 2D NMR techniques. IR spectroscopy: ATR technique.

Mass spectrometry: Exact masses (APCI and LC-MS): Deviations of the found exact masses from the calculated exact masses: 5 mDa or less. LC-HRMS: column: Kinetex^TM^, 2.6 μm, C18, 100 Å; 50 mm/2.1 mm, guard column: Security Guard Standard C18 Cartridge, 4 mm/2mm, temperature: 30 °C, solvents: A: acetonitrile-NH_4_HCOO (10 mM) = 10:90 + 0.1% (*v/v*) HCO_2_H, B: acetonitrile-NH_4_HCO_2_ (10 mM) = 90:10 + 0.1% (v/v) HCO_2_H, gradient elution: (A%): 0–5 min: gradient from 100% to 0%, flow rate: 0.4 mL/min, 5–6.5 min: 0%, flow rate: 0.4 mL/min, 6.5–7 min: gradient from 0% to 100%, flow rate: 0.4 mL/min, 10 min: 100%, flow rate: 0.6 mL/min, injection volume: 0.5–1 μL, sample temperature: 5 °C, UV detection wavelength: 200–350 nm.

HPLC for determination of compound purity (method 1): column: LiChrospher^®^ 60 RP-select B (5 µm), LiChroCART^®^ 250-4 mm cartridge; guard Column: LiChrospher^®^ 60 RP-select B (5 µm), LiCroCART^®^ 4-4 mm cartridge (No.: 1.50963.0001) using manu-CART^®^ NT cartridge holder; solvents: A: water with 0.05% (v/v) trifluoroacetic acid; B: acetonitrile with 0.05% (v/v) trifluoroacetic acid; gradient elution: (A %): 0–4 min: 90%, 4–29 min: gradient from 90% to 0%, 29–31 min: 0%, 31–31.5 min: gradient from 0% to 90%, 31.5–40 min: 90%; flow rate: 1.0 mL/min; injection volume: 5.0 µL; UV detection wavelength: 210 nm; stop time: 30.0 min. Chiral HPLC for determination of enantiomeric purity (method 2): UV detection wavelength: 210 nm; stop time: 30.0 min; parentheses include: column, solvent, flow rate, injection volume.

#### 2.1.2. Synthetic Procedures

*tert-Butyl-4-hydroxy-4-(2-vinylphenyl)piperidine-1-carboxylate* (**6**)

2-Bromostyrene (**5**, 3.1 g, 16.9 mmol) was dissolved in THF (125 mL). The solution was cooled to −78 °C under N_2_ atmosphere. A solution of *n*-butyllithium in hexanes (15 mL, 24 mmol) was added dropwise and the mixture was stirred for 15 min. Then *tert*-butyl 4-oxopiperidine-1-carboxylate (4 m, 4.1 g, 20.6 mmol), dissolved in THF (50 mL), was added and the mixture was stirred at −78 °C for 2.5 h. Then the solution was warmed to ambient temperature. A solution of LiBH_4_ in THF (5 mL, 20 mmol) was added dropwise and the mixture was stirred for 1 h at ambient temperature. The reaction was stopped by the addition of water and a 1 M aqueous solution of HCl. After separation of the layers, the aqueous layer was extracted with CH_2_Cl_2_ (3×). The combined organic layers were dried (Na_2_SO_4_), filtered and the solvent was removed *in vacuo*. The crude product was purified by flash column chromatography (Ø = 8 cm, h = 16 cm, cyclohexane-ethyl acetate = 9:1, V = 100 mL) to give **6** as a colorless solid (R_f_ = 0.34, cyclohexane-ethyl acetate = 8:2), mp 104 °C, yield 3.83 g (75%). C_18_H_25_NO_3_ (303.4 g/mol). Purity (HPLC method 1): 99.4%, t_R_ = 20.4 min. Exact mass (APCI): *m/z* = 304.1882 (calcd. 304.1907 for C_18_H_26_NO_3_ [M+H]^+^). ^1^H-NMR (CDCl_3_): δ (ppm) = 1.47 (s, 9H, CO_2_C(C*H*_3_)_3_), 1.64 (s br, 1H, O*H*), 1.93–2.10 (m, 4H, N(CH_2_C*H*_2_)_2_), 3.32–3.36 (m, 2H, N(C*H*_2_CH_2_)_2_), 3.89–4.13 (m, 2H, N(C*H*_2_CH_2_)_2_), 5.28 (dd, *J* = 10.9/1.8 Hz, 1H, HC=C*H*_2_), 5.51 (dd, *J* = 17.4/1.8 Hz, 1H, HC=C*H*_2_), 7.24–7.31 (m, 2H, 3-H_arom._, 4-H_arom._), 7.34–7.39 (m, 1H, 6-H_arom._)_,_ 7.45–7.50 (m, 1H, 5-H_arom._)_,_ 7.65 (dd, *J* = 17.4/10.9 Hz, 1H, *H*C=CH_2_). ^13^C NMR (CDCl_3_): δ (ppm) = 28.6 (3C, CO_2_C(*C*H_3_)_3_), 37.4 (br, 2C, N(CH_2_*C*H_2_)_2_), 39.5 (br, 1C, N(*C*H_2_CH_2_)_2_), 40.3 (br, 1C, N(*C*H_2_CH_2_)_2_), 72.5 (1C, Ar*C*OH ), 79.6 (1C, CO_2_*C*(CH_3_)_3_), 115.7 (1C, HC=*C*H_2_), 124.9 (1C, C-6_arom._), 127.7 (1C, C-4_arom._), 127.8 (1C, C-3_arom._), 129.1 (1C, C-5_arom._), 137.9 (1C, C-2_arom._), 138.0 (1C, H*C*=CH_2_), 143.8 (1C, C-1_arom._), 155.0 (1C, *C*O_2_C(CH_3_)_3_). FT-IR (neat): 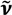
(cm^−1^) = 3387 (O-H), 2967, 2932, (C-H), 1655 (C=O), 756 (1,2-disubst. arom.).

*tert*-Butyl (*R*)-4-[2-(1,2-dihydroxyethyl)phenyl]-4-hydroxypiperidine-1-carboxylate (**(*R*)-7**)

AD-mix-β (27.1 g) was added to a mixture of *tert*-butyl alcohol (600 mL) and water (600 mL). The mixture was cooled to 0 °C, **6** (5.9 g, 19.5 mmol) was added and the reaction mixture was stirred at 0 °C for 3 d. Then sodium sulfite (29 g) was added and the mixture was allowed to warm to room temperature and stirred for 20 min. Ethyl acetate was added to the reaction mixture, and after separation of the layers, the aqueous layer was extracted with ethyl acetate (3×). The combined organic layers were dried (Na_2_SO_4_), filtered and the solvent was removed *in vacuo*. The crude product was purified by flash column chromatography (Ø = 8 cm, h = 18 cm, cyclohexane-ethyl acetate = 1:2 → ethyl acetate, V = 100 mL) to give **(*R*)-7** as a colorless solid (R_f_ = 0.11, cyclohexane-ethyl acetate = 5:5), mp 93 °C, yield 5.4 g (82%). C_18_H_27_NO_5_ (337.4 g/mol). Specific rotation: 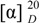: = ‒24.5 (3.5; CH_2_Cl_2_). Purity (HPLC method 1): 99.5%, t_R_ = 15.3 min.

*tert*-Butyl (*S*)-4-(2-[1,2-dihydroxyethyl)phenyl]-4-hydroxypiperidine-1-carboxylate (**(*S*)-7**)

AD-mix-α (15.2 g) was added to a mixture of *tert*-butyl alcohol (325 mL) and water (325 mL). The mixture was cooled to 0 °C, **6** (4.5 g, 14.9 mmol) was added and the reaction mixture was stirred overnight at 0 °C. Then methanesulfonamide (1.0 g, 10.5 mmol) was added and the mixture was stirred overnight at ambient temperature. Then sodium sulfite (16.2 g) was added and the mixture stirred for 30 min. Ethyl acetate was added to the reaction mixture, and after separation of the layers, the aqueous layer was extracted with ethyl acetate (3×). The combined organic layers were washed with a 2 M aqueous solution of NaOH, dried (Na_2_SO_4_), filtered and the solvent was removed *in vacuo*. The crude product was purified by flash column chromatography (Ø = 8 cm, h = 15 cm, cyclohexane-ethyl acetate = 1:2 → ethyl acetate, V = 100 mL) to give **(*S*)-7** as a colorless solid (R_f_ = 0.11, cyclohexane:ethyl acetate = 5:5), mp 87 °C, yield 2.7 (74%) C_18_H_27_NO_5_ (337.4 g/mol). Specific rotation: 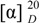: = +25.8 (3.7; CH_2_Cl_2_). Purity (HPLC method 1): 95.5%, t_R_ = 15.5 min.

Spectroscopic data for **(*R*)-7** and **(*S*)-7**

LC-HRMS: *m/z* = 360.1800 (calcd. 360.1781 for C_18_H_27_NNaO_5_ [M+Na]^+^). ^1^H-NMR (CDCl_3_): δ (ppm) = 1.47 (s, 9H, CO_2_(C*H*_3_)_3_), 1.82–2.11 (m, 4H, N(CH_2_C*H*_2_)_2_), 3.24 (t, *J* = 12.6 Hz, 2H, N(C*H*_2_CH_2_)_2_), 3.78 (dd, *J* = 10.9/4.2 Hz, 1H, HOCHC*H*_2_OH), 3.86 (dd, *J* = 10.9/7.7 Hz, 1H, HOCHC*H*_2_OH), 3.95–4.05 (m, 2H, N(C*H*_2_CH_2_)_2_), 5.64 (dd, *J* = 7.7/4.2 Hz, 1H, HOC*H*CH_2_OH), 7.23–7.32 (m, 3H, H_arom._), 7.48–7.52 (m, 1H, H_arom._). Signals for the OH protons are not visible in the spectrum. ^13^C-NMR (CDCl_3_): δ (ppm) = 28.6 (3C, CO_2_C(*C*H_3_)_3_), 38.3 (br, 1C, N(CH_2_*C*H_2_)_2_), 38.6 (br, 1C, N(CH_2_*C*H_2_)_2_), 39.4 (br, 1C, N(*C*H_2_CH_2_)_2_), 40.0 (br, 1C, N(*C*H_2_CH_2_)_2_), 68.0 (1C, HOCH*C*H_2_OH), 72.1 (1C, HO*C*HCH_2_OH), 72.7 (1C, Ar*C*OH), 79.8 (1C, CO_2_*C*(CH_3_)_3_), 125.6 (1C, C_arom._), 127.8 (1C, C_arom._), 127.9 (1C, C_arom._), 129.1 (1C, C_arom._), 139.5 (1C, C_arom._), 145.0 (1C, C_arom._), 155.1 (1C, *C*O_2_C(CH_3_)_3_). IR (neat): 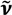 (cm^−1^) = 3387 (O-H), 2974, 2925, (C-H), 1663 (C=O), 1246, 1161 (C-O-C ester), 756 (1,2-disubst. arom.).

*tert*-Butyl (*S*)-3-[(tosyloxy)methyl]-3*H*-spiro[[2]benzofuran-1,4′-piperidine]-1′-carboxylate (**(*S*)-8**)

**(*R*)-7** (98 mg, 0.29 mmol) was dissolved in CH_2_Cl_2_ (10 mL). 4-Dimethylaminopyridine (12 mg, 0.10 mmol), triethylamine (210 μL, 1.5 mmol) and 4-toulenesulfonyl chloride (110 mg, 0.58 mmol) were added and the mixture was stirred for 3 h at ambient temperature. Then water was added and after separation of the layers, the aqueous layer was extracted with CH_2_Cl_2_ (3×). The combined organic layers were dried (Na_2_SO_4_), filtered and the solvent was removed *in vacuo*. The crude product was purified by flash column chromatography (Ø = 2 cm, h = 16 cm, cyclohexane-ethyl acetate = 9:1, V = 10 mL) to give **(*S*)-8** as a colorless oil (R_f_ = 0.28, cyclohexane-ethyl acetate = 8:2), mp 124 °C, yield 69 mg (50%). C_25_H_31_NO_6_S (473.6 g/mol). Specific rotation: 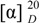: = +19.2 (7.6; CH_2_Cl_2_). Purity (HPLC method 1): 98.8%, t_R_ = 22.8 min. Exact mass (APCI): *m/z* = 474.1969 (calcd. 474.1945 for C_25_H_32_NO_6_S [M+H]^+^). ^1^H-NMR (CDCl_3_): δ (ppm) = 1.48 (s, 9H, CO_2_C(C*H*_3_)_3_), 1.51–1.70 (m, 2H, N(CH_2_C*H*_2_)_2_), 1.70 (td, *J* = 13.1/4.8 Hz, 1H, N(CH_2_C*H*_2_)_2_), 1.82 (td, *J* = 13.1/4.8 Hz, 1H, N(CH_2_C*H*_2_)_2_), 2.44 (s, 3H, C*H*_3_), 3.02 (td, *J* = 12.9/2.9 Hz, 1H, N(C*H*_2_CH_2_)_2_), 3.12 (td, *J* = 12.9/2.9 Hz, 1H, N(C*H*_2_CH_2_)_2_), 3.95–4.07 (m, 2H, N(C*H*_2_CH_2_)_2_), 4.15 (dd, *J* = 10.2/5.3 Hz, 1H, C*H*_2_OTos), 4.24 (dd, *J* = 10.2/4.1 Hz, 1H, C*H*_2_OTos), 5.37 (t, *J* = 4.7 Hz 1H, ArC*H*O), 7.04–7.08 (m, 1H, H_arom._), 7.12–7.17 (m, 1H, H_arom._), 7.24–7.35 (m, 4H, 3-H_tosyl_, 5-H_tosyl_, H_arom._ (2H)), 7.71–7.75 (m, 2H, 2-H_tosyl_, 6-H_tosyl_). ^13^C-NMR (CDCl_3_): δ (ppm) = 21.8 (1C, *C*H_3_), 28.6 (3C, CO_2_C(*C*H_3_)_3_), 37.2 (1C, N(CH_2_*C*H_2_)_2_), 37.9 (1C, N(CH_2_*C*H_2_)_2_), 40.4 (1C, N(*C*H_2_CH_2_)_2_), 40.6 (1C, N(*C*H_2_CH_2_)_2_), 72.2 (1C, *C*H_2_OTos), 79.4 (1C, Ar*C*HO), 79.6 (1C, CO_2_*C*(CH_3_)_3_), 85.4 (1C, Ar*C*O), 121.1 (1C, C_arom._), 122.0 (1C, C_arom._), 128.1 (2C, C-2_tosyl_, C-6_tosyl_), 128.3 (1C, C_arom._), 128.8 (1C, C_arom._), 130.0 (2C, C-3_tosyl_, C-5_tosyl_), 133.0 (1C, C_arom._), 136.9 (1C, C_arom._), 145.1 (1C, C_arom._), 145.8 (1C, C_arom._), 155.1 (1C, *C*O_2_C(CH_3_)_3_). IR (neat): 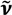 (cm^−1^) = 2978, 2870 (C-H), 1686 (C=O), 1362 (O=S=O), 1234, 1173 (C-O-C, ester), 1069 (C-O-C, ether), 768 (1,2-disubst. arom.).

*tert*-Butyl (*R*)-4-hydroxy-3,4-dihydrospiro[[2]benzopyran-1,4′-piperidine]-1′-carboxylate (**(*R*)-11**)

**(*R*)-7** (908 mg, 2.7 mmol) was dissolved in THF (25 mL). Dibutyltin oxide (75 mg, 0.30 mmol), triethylamine (744 μL, 5.4 mmol) and toluene-4-sulfonyl chloride (1.0 g, 5.3 mmol) were added and the mixture was stirred for 3 h at ambient temperature. Then water and CH_2_Cl_2_ were added. After separation of the layers, the aqueous layer was extracted with CH_2_Cl_2_ (3×). The combined organic layers were dried (Na_2_SO_4_), filtered and the solvent was removed *in vacuo*. The crude product was purified by flash column chromatography (Ø = 5 cm, h = 15 cm, cyclohexane-ethyl acetate = 3:1, V = 30 mL) to give **(*R*)-11** as a colorless solid (R_f_ = 0.25, cyclohexane-ethyl acetate = 2:1), mp 161 °C, yield 538 mg (62%). C_18_H_25_NO_4_ (319.4 g/mol). Specific rotation: 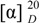: = −4.9 (11.0; CH_2_Cl_2_). Purity (HPLC method 1): 96.0%, t_R_ = 18.6 min. Enantiomeric ratio (HPLC method 2, Daicel Chiralpak AD-H, 5 μm, 250 mm/4.6 mm, isohexane:methanol = 95:5, flow rate: 1.0 mL/min, injection volume: 10 μL): (*R*):(*S*) = 92.5:7.5, t_R_ = 11.4 min.

*tert*-Butyl (*S*)-4-hydroxy-3,4-dihydrospiro[[2]benzopyran-1,4′-piperidine]-1′-carboxylate (**(*S*)-11**)

**(*S*)-7** (996 mg, 3.0 mmol) was dissolved in THF (15 mL). Dibutyltin oxide (86 mg, 0.35 mmol), triethylamine (2.0 mL, 14.8 mmol) and toluene-4-sulfonyl chloride (2.1 g, 6.3 mmol) were added and the mixture was stirred for 3 days at ambient temperature. Then water and CH_2_Cl_2_ were added. After separation of the layers, the aqueous layer was extracted with CH_2_Cl_2_ (3×). The combined organic layers were dried (Na_2_SO_4_), filtered and the solvent was removed *in vacuo*. The crude product was purified by flash column chromatography (Ø = 5 cm, h = 15 cm, cyclohexane-ethyl acetate = 3:1, V = 65 mL) to give **(*S*)-11** as a colorless solid (R_f_ = 0.25, cyclohexane-ethyl acetate = 2:1), mp 158 °C, yield 447 mg (47%). C_18_H_25_NO_4_ (319.4 g/mol). Specific rotation: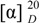: = +5.2 (4.4; CH_2_Cl_2_). Purity (HPLC method 1): 98.3%, t_R_ = 18.3 min. Enantiomeric ratio (HPLC method 2, Daicel Chiralpak AD-H, 5 μm, 250 mm/4.6 mm, isohexane:methanol = 95:5, flow rate: 1.0 mL/min, injection volume: 10 μL): (*R*):(*S*) = 11.4:88.6, t_R_ = 20.7 min.

Spectroscopic data for **(*R*)-11** and **(*S*)-11**

Exact mass (APCI): *m/z* = 320.1894 (calcd. 320.1856 for C_18_H_26_NO_4_ [M+H]^+^). ^1^H-NMR (CDCl_3_): δ (ppm) = 1.49 (s, 9H, CO_2_(C*H*_3_)_3_), 1.69–1.83 (m, 2H, N(CH_2_C*H*_2_)_2_), 1.90–2.05 (m, 2H, N(CH_2_C*H*_2_)_2_), 3.11 (t, *J* = 13.0 Hz, 1H, N(C*H*_2_CH_2_)_2_), 3.22 (t, *J* = 13.0 Hz, 1H, N(C*H*_2_CH_2_)_2_), 3.92 (dd, *J* = 12.1/3.3 Hz, 1H, HOCHC*H*_2_O), 3.98 (dd, *J* = 12.1/2.7 Hz, 1H, HOCHC*H*_2_O), 3.99–4.07 (m, 2H, N(C*H*_2_CH_2_)_2_), 4.54 (t, *J* = 3.0 Hz, 1H, HOC*H*CH_2_O), 7.10 (dd, *J* = 7.4/1.6 Hz, 1H, 8-H_arom._), 7.25–7.34 (m, 2H, 6-H_arom._, 7-H_arom._), 7.42 (dd, *J* = 7.2/1.8 Hz, 1H, 5-H_arom._). A signal for the OH proton is not visible in the spectrum. ^13^C-NMR (CDCl_3_): δ (ppm) = 28.6 (3C, CO_2_C(*C*H_3_)_3_), 34.3 (br, 1C, N(CH_2_*C*H_2_)_2_), 37.6 (br, 1C, N(CH_2_*C*H_2_)_2_), 39.5 (br, 1C, N(*C*H_2_CH_2_)_2_), 40.1 (br, 1C, N(*C*H_2_CH_2_)_2_), 64.8 (1C, HOCH*C*H_2_O), 66.0 (1C, HO*C*HCH_2_O), 73.8 (1C, Ar*C*O), 79.7 (1C, CO_2_*C*(CH_3_)_3_), 125.2 (1C, C-8_arom._), 127.3 (1C, C-6_arom._), 128.6 (1C, C-7_arom._), 129.1 (1C, C-5_arom._), 135.3 (1C, C-8a_arom._), 141.2 (1C, C-4a_arom._), 155.1 (1C, *C*O_2_C(CH_3_)_3_). IR (neat): 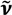 [cm^−1^] = 3314 (O-H), 2974, 2928 (C-H), 1686 (C=O), 1169 (C-O-C ether), 768 (1,2-disubst. arom.).

(*R*)-1′-Benzyl-3,4-dihydrospiro[[2]benzopyran-1,4′-piperidin]-4-ol (**(*R*)-12**)

**(*R*)-11** (105 mg, 0.33 mmol) was dissolved in CH_2_Cl_2_ (4 mL). The solution was cooled to 0 °C. Then trifluoroacetic acid (200 μL) was added and the mixture was stirred for 3.5 h at 0 °C. Then a 2 m aqueous solution of sodium hydroxide (4 mL) was added, and after separation of the layers, the aqueous layer was extracted with CH_2_Cl_2_ (3×). The combined organic layers were dried (Na_2_SO_4_), filtered and the solvent was removed *in vacuo*. The residue was dissolved in CH_2_Cl_2_ (5 mL), benzaldehyde (35 μL, 0.35 mmol) and sodium triacetoxyborohydride (85 mg, 0.40 mmol) were added and the mixture was stirred overnight at ambient temperature. The reaction was stopped by the addition of a 2 M aqueous solution of sodium hydroxide, and after separation of the layers, the aqueous layer was extracted with CH_2_Cl_2_ (4×). The combined organic layers were dried (Na_2_SO_4_), filtered and the solvent was removed *in vacuo*. The crude product was purified by flash column chromatography (Ø = 1.5 cm, h = 16 cm, cyclohexane-ethyl acetate = 5:1 + 1% *N,N*-dimethylethylamine, V = 5 mL) to give **(*R*)-12** as a colorless solid (R_f_ = 0.14, cyclohexane-ethyl acetate = 5:5), mp 55 °C), yield 57 mg (56%). C_20_H_23_NO_2_ (309.4 g/mol). Specific rotation: 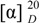: = −8.4 (2.3; CH_2_Cl_2_). Purity (HPLC method 1): 95.3%, t_R_ = 13.5 min. Enantiomeric ratio (HPLC method 2, Daicel Chiralpak AD-H, 5 μm, 250 mm/4.6 mm, isohexane-isopropanol = 95:5, flow rate: 1.0 mL/min, injection volume: 10 μL): (*R*):(*S*) = 96.1:3.9, t_R_ = 9.7 min).

(*S*)-1′-Benzyl-3,4-dihydrospiro[2-benzopyran-1,4′-piperidin]-4-ol (**(*S*)-12**)

**(*R*)-11** (56 mg, 0.18 mmol) was dissolved in CH_2_Cl_2_ (10 mL). Trifluoroacetic acid (200 μL) was added and the mixture was stirred overnight at ambient temperature. Then water was added, and after separation of the layers, the aqueous layer was extracted with CH_2_Cl_2_ (3×). The combined organic layers were dried (Na_2_SO_4_), filtered and the solvent was removed *in vacuo*. The residue was dissolved in CH_2_Cl_2_ (10 mL), benzaldehyde (50 μL, 0.45 mmol) and sodium triacetoxyborohydride (50 mg, 0.24 mmol) were added and the mixture was stirred for 5.5 h at ambient temperature. Then benzaldehyde (50 μL, 0.35 mmol) and sodium triacetoxyborohydride (60 mg, 0.28 mmol) were added and the mixture was stirred overnight at ambient temperature). The reaction was stopped by the addition of a 2 M aqueous solution of sodium hydroxide, and after separation of the layers, the aqueous layer was extracted with CH_2_Cl_2_ (3×). The combined organic layers were dried (Na_2_SO_4_), filtered and the solvent was removed *in vacuo*. The crude product was purified by flash column chromatography (Ø = 1 cm, h = 15 cm, cyclohexane-ethyl acetate = 5:1 + 1% *N,N*-dimethylethylamine, V = 5 mL) to give **(*S*)-12** as a colorless solid (R_f_ = 0.14, cyclohexane-ethyl acetate = 5:5) mp 53 °C, yield 12 mg (22%). C_20_H_23_NO_2_ (309.4 g/mol). Specific rotation: 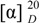: = +8.8 (2.2; CH_2_Cl_2_). Purity (HPLC method 1): 98.3%, t_R_ = 13.4 min. Enantiomeric ratio (HPLC method 2, Daicel Chiralpak AD-H, 5 μm, 250 mm/4.6 mm, isohexane:isopropanol = 95:5, flow rate: 1.0 mL/min, injection volume: 10 μL): (*R*):(*S*) = 11.9:88.1, t_R_ = 12.9 min.

Spectroscopic data for **(*R*)-12** and **(*S*)-12**

Exact mass (APCI): *m/z* = 310.1802 (calcd. 310.1802 for C_20_H_24_NO_2_ [M+H]^+^). ^1^H-NMR (CDCl_3_): δ (ppm) = 1.78–1.98 (m, 3H, N(CH_2_C*H*_2_)_2_), 2.18 (td, *J* = 13.1/4.6 Hz, 1H, N(CH_2_C*H*_2_)_2_), 2.39 (dd, *J* = 11.9/3.0 Hz, 1H, N(C*H*_2_CH_2_)_2_), 2.46–2.54 (m, 1H, N(C*H*_2_CH_2_)_2_), 2.75 (t, *J* = 13.1/Hz, 2H, N(C*H*_2_CH_2_)_2_), 3.58 (s, 2H, NC*H*_2_Ph), 3.89 (dd, *J* = 12.1/3.3 Hz, 1H, CHC*H*_2_O), 3.97 (dd, *J* = 12.1/2.6 Hz, 1H, CHC*H*_2_O), 4.51 (t, *J* = 2.9 Hz, 1H, C*H*CH_2_O), 7.20–7.41 (m, 9H, H_arom._). A signal for the OH proton is not visible in the spectrum. ^13^C-NMR (CDCl_3_): δ (ppm) = 34.7 (1C, N(CH_2_*C*H_2_)_2_), 38.0 (1C, N(CH_2_*C*H_2_)_2_), 49.3 (1C, N(*C*H_2_CH_2_)_2_), 49.3 (1C, N(*C*H_2_CH_2_)_2_), 63.5 (1C, N*C*H_2_Ph), 64.5 (1C, CH*C*H_2_O ), 66.1 (1C, *C*HCH_2_O), 73.9 (1C, Ar*C*O), 125.3 (1C, C_arom._), 127.0 (1C, C_arom._), 127.1 (1C, C_arom._), 128.3 (2C, C_arom._), 128.5 (1C, C_arom._), 128.9 (1C, C_arom._), 129.4 (2C, C_arom._), 135.5 (1C, C_arom._), 138.6 (1C, C_arom._), 141.8 (1C, C_arom._). IR (neat): 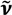
(cm^−1^) = 3329 (O-H), 2924, 2817 (C-H), 1072 (C-O-C), 733 (1,2-disubst. arom.), 698 (monosubst. arom.).

*tert*-Butyl (*R*)-4-methoxy-3,4-dihydrospiro[[2]benzopyran-1,4′-piperidine]-1′-carboxylate (**(*R*)-13**)

**(*R*)-10** (450 mg, 1.4 mmol) was dissolved in THF (12 mL). NaH (60% dispersion in paraffin liquid, 112 mg, 2.8 mmol) was added and the mixture was stirred for 1 h at ambient temperature. Then iodomethane (176 μL, 2.8 mmol) was added dropwise and the mixture was stirred for 1 h at ambient temperature. The solvent was removed *in vacuo*. The crude product was purified by flash column chromatography (Ø = 2.5 cm, h = 16.5 cm, cyclohexane-ethyl acetate = 9:1, V = 10 mL) to give **(*R*)-13** as a colorless oil (R_f_ = 0.28, cyclohexane-ethyl acetate = 8:2), yield 465 mg (99%). C_19_H_27_NO_4_ (333.4 g/mol). Specific rotation:
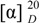
= −8.0 (4.4; CH_2_Cl_2_). Purity (HPLC method 1): 96.6%, t_R_ = 20.4 min.

*tert*-Butyl (*S*)-4-methoxy-3,4-dihydrospiro[2-benzopyran-1,4′-piperidine]-1′-carboxylate (**(*S*)**-**13**)

**(*S*)-10** (180 mg, 0.56 mmol) was dissolved in THF (2.5 mL). NaH (60% dispersion in paraffin liquid, 50 mg, 1.3 mmol) was added and the mixture was stirred for 1 h at ambient temperature. Then iodomethane (77 μL, 1.2 mmol) was added dropwise and the mixture was stirred overnight at ambient temperature. The solvent was removed *in vacuo*. The crude product was purified by flash column chromatography (Ø = 2 cm, h = 15 cm, cyclohexane:ethyl acetate = 9:1, V = 10 mL) to give **(*S*)-13** as a pale yellow oil (R_f_ = 0.28, cyclohexane:ethyl acetate = 8:2), yield 128 mg (69%). C_19_H_27_NO_4_ (333.4 g/mol). Specific rotation: 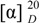: = +7.4 (7.9; CH_2_Cl_2_). Purity (HPLC method 1): 97.7%, t_R_ = 20.4 min.

Spectroscopic data for **(*R*)-13** and **(*S*)-13**

Exact mass (APCI): *m/z* = 334.2009 (calcd. 334.2013 for C_19_H_28_NO_4_ [M+H]^+^). ^1^H-NMR (CDCl_3_): δ (ppm) = 1.49 (s, 9H, CO_2_(C*H*_3_)_3_), 1.75 (td, *J* = 13.2/4.9 Hz, 1H, N(CH_2_C*H*_2_)_2_), 1.84–1.99 (m, 3H, N(CH_2_C*H*_2_)_2_), 3.03–3.28 (m, 2H, N(C*H*_2_CH_2_)_2_), 3.50 (s, 3H, OC*H*_3_), 3.93–4.06 (m, 4H, N(C*H*_2_CH_2_)_2_ (2), CHC*H*_2_O (2)), 4.19 (t, *J* = 3.4 Hz, 1H, C*H*CH_2_O), 7.11 (dd, *J* = 7.7/1.4 Hz, 1H, H_arom._), 7.25 (td, *J* = 7.4/1.4 Hz, 1H, H_arom._), 7.30 (td, *J* = 7.4/1.6 Hz, 1H, H_arom._), 7.37 (dd, *J* = 7.4/1.6 Hz, 1H, H_arom._). ^13^C-NMR (CDCl_3_): δ (ppm) = 28.6 (3C, CO_2_C(*C*H_3_)_3_), 34.9 (br, 1C, N(CH_2_*C*H_2_)_2_), 37.0 (br, 1C, N(CH_2_*C*H_2_)_2_), 39.4 (br, 1C, N(*C*H_2_CH_2_)_2_), 40.2 (br, 1C, N(*C*H_2_CH_2_)_2_), 56.9 (1C, O*C*H_3_), 61.6 (1C, CH*C*H_2_O), 73.5 (1C, Ar*C*O), 74.0 (1C, *C*HCH_2_O), 79.5 (1C, CO_2_*C*(CH_3_)_3_), 125.1 (1C, C_arom._), 126.7 (1C, C_arom._), 128.4 (1C, C_arom._), 129.1 (1C, C_arom._), 132.8 (1C, C_arom._), 141.8 (1C, C_arom._), 155.0 (1C, *C*O_2_C(CH_3_)_3_). IR (neat): 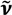
(cm^−1^) = 2970, 2928 (_C-H_), 1686 (_C=O_), 1084 (_C-O-C ether_), 756 (1,2-disubst. arom.).

*tert*-Butyl (*R*)-4-ethoxy-3,4-dihyrospiro[[2]benzopyran-1,4′-piperidine]-1′-carboxylate (**(*R*)-14**)

**(*R*)-10** (210 mg, 0.66 mmol) was dissolved in THF (15 mL). NaH (60% dispersion in paraffin liquid, 80 mg, 2.0 mmol) was added and the mixture was stirred for 1 h at ambient temperature. Then iodoethane (0.53 mL, 6.6 mmol) was added dropwise and the mixture was stirred for 2.5 h at ambient temperature. A 1 m solution of lithium bis(trimethylsilyl)amide (4.5 mL) was added and the mixture was heated to reflux overnight. The mixture was allowed to cool to ambient temperature and stirred overnight. Then water and CH_2_Cl_2_ were added. After separation of the layers, the aqueous layer was extracted with CH_2_Cl_2_ (3×). The combined organic layers were dried (Na_2_SO_4_), filtered and the solvent removed *in vacuo*. The crude product was purified by flash column chromatography (Ø = 2 cm, h = 15 cm, cyclohexane-ethyl acetate = 9:1, V = 10 mL) to give **(*R*)-14** as a pale yellow oil (R_f_ = 0.15, cyclo-hexane-ethyl acetate = 9:1), yield 66 mg (29%). C_20_H_29_NO_4_ (347.4 g/mol). Specific rotation: 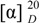: = −2.2 (3.2; CH_2_Cl_2_). Purity (HPLC method 1): 96.3%, t_R_ = 21.3 min.

*tert*-Butyl (*S*)-4-ethoxy-3,4-dihydrospiro[[2]benzopyran-1,4′-piperidine]-1′-carboxylate (**(*S*)-14**)

**(*S*)-10** (200 mg, 0.63 mmol) was dissolved in THF (5 mL). A 1 m solution of lithium bis(trimethylsilyl)amide (6.3 mL) was added and the mixture was stirred for 1 h at ambient temperature. Then iodoethane (500 μL, 6.3 mmol) was added dropwise and the mixture was stirred for 16 h at ambient temperature. NaH (60% dispersion in paraffin liquid, 250 mg, 6.3 mmol) and iodoethane (500 μL, 6.3 mmol) were added and the mixture was heated to reflux overnight. The mixture was allowed to cool to ambient temperature and stirred for 3 days. Then water was added. After separation of the layers, the aqueous layer was extracted with ethyl acetate (3×). The combined organic layers were dried (Na_2_SO_4_), filtered and the solvent was removed *in vacuo*. The crude product was purified by flash column chromatography three times (1. Ø = 2 cm, h = 15 cm, cyclohexane-ethyl acetate = 9:1, V = 10 mL; 2. Ø = 1.5 cm, h = 15 cm, cyclohexane-ethyl acetate = 9:1, V = 5 mL; 3. Ø = 1.5 cm, h = 15 cm, cyclohexane-ethyl acetate = 95:5, V = 5 mL) to give **(*S*)-14** as a pale yellow oil (R_f_ = 0.15, cyclohexane-ethyl acetate = 9:1), yield 110 mg (50%). C_20_H_29_NO_4_ (347.4 g/mol). Specific rotation: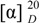: = +2.4 (3.9; CH_2_Cl_2_). Purity (HPLC method 1): 98.4%, t_R_ = 21.5 min.

Spectroscopic data for **(*R*)-14** and **(*S*)-14**

Exact mass (APCI): *m/z* = 348.2199 (calcd. 348.2169 for C_20_H_30_NO_4_ [M+H]^+^). ^1^H-NMR (CDCl_3_): δ (ppm) = 1.28 (t, *J* = 7.0 Hz, 3H, OCH_2_C*H*_3_), 1.49 (s, 9H, CO_2_(C*H*_3_)_3_), 1.76–1.86 (m, 2H, N(CH_2_C*H*_2_)_2_), 1.86–1.96 (m, 2H, N(CH_2_C*H*_2_)_2_), 3.04–3.26 (m, 2H, N(C*H*_2_CH_2_)_2_), 3.64–3.78 (m, 2H, OC*H*_2_CH_3_), 3.86–4.08 (m, 2H, N(C*H*_2_CH_2_)_2_), 3.89 (dd, *J* = 12.0/5.4 Hz, 1H, CHC*H*_2_O), 4.00 (dd, *J* = 12.0/3.7 Hz, 1H, CHC*H*_2_O), 4.35 (t, *J* = 4.5 Hz, 1H, C*H*CH_2_O), 7.08 (dd, *J* = 7.2/2.0 Hz, 1H, H_arom._), 7.20–7.32 (m, 2H, H_arom._), 7.42 (dd, *J* = 6.9/2.3 Hz, 1H, H_arom._). ^13^C-NMR (CDCl_3_): δ (ppm) = 15.9 (1C, OCH_2_*C*H_3_), 28.7 (3C, CO_2_C(*C*H_3_)_3_), 35.8 (br, 1C, N(CH_2_*C*H_2_)_2_), 36.4 (br, 1C, N(CH_2_*C*H_2_)_2_), 39.5 (br, 1C, N(*C*H_2_CH_2_)_2_), 40.3 (br, 1C, N(*C*H_2_CH_2_)_2_), 62.1 (1C, CH*C*H_2_O), 64.9 (1C, O*C*H_2_CH_3_), 72.4 (1C, *C*HCH_2_O), 73.7 (1C, CO_2_*C*(CH_3_)_3_), 79.6 (1C, Ar*C*O), 125.1 (1C, C_arom._), 126.8 (1C, C_arom._), 128.1 (1C, C_arom._), 128.5 (1C, C_arom._), 134.0 (1C, C_arom._), 141.8 (1C, C_arom._), 155.1 (1C, *C*O_2_C(CH_3_)_3_). IR (neat): 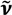
(cm^−1^) = 2970, 2928 (C-H), 1690 (C=O), 1092 (C-O-C ether), 756 (1,2-disubst. arom.).

(*R*)-1′-Benzyl-4-methoxy-3,4-dihydrospiro[[2]benzopyran-1,4′-piperidine] (**(*R*)-15**)

(*R*)-**13** (360 mg, 1.1 mmol) was dissolved in CH_2_Cl_2_ (5 mL). The solution was cooled to 0 °C. Trifluoroacetic acid (0.7 mL) was added and the mixture was stirred for 2 h at 0 °C. Then a 2 M aqueous solution of NaOH was added. After separation of the layers, the aqueous layer was extracted with CH_2_Cl_2_ (3×). The combined organic layers were dried (Na_2_SO_4_), filtered and the solvent was removed *in vacuo*. The residue was dissolved in CH_2_Cl_2_ (5 mL). Benzaldehyde (30 μL, 0.30 mmol) and sodium triacetoxyborohydride (76 mg, 0.36 mmol) were added and the mixture was stirred for 26 h at ambient temperature. Then a 2 M aqueous solution of NaOH (3 mL) and water (3 mL) were added. After separation of the layers, the aqueous layer was extracted with CH_2_Cl_2_ (3×). The combined organic layers were dried (Na_2_SO_4_), filtered and the solvent was removed *in vacuo*. The crude product was purified by flash column chromatography (Ø = 0.75 cm, h = 15 cm, cyclohexane-ethyl acetate = 4:1, V = 5 mL) to give **(*R*)-15** as a colorless oil (R_f_ = 0.27, cyclohexane-ethyl acetate = 5:5), yield 31 mg (9%). C_21_H_25_NO_2_ (323.4 G/mol). Specific rotation: 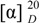: = −8.6 (2.8; CH_2_Cl_2_). Purity (HPLC method 1): 98.2%, t_R_ = 15.8 min. Enantiomeric ratio (HPLC method 2, Daicel Chiralpak IB, 5 μm, 250 mm/4.6 mm, isohexane-methanol = 97:3, flow rate: 1.0 mL/min, injection volume: 5 μL): (*R*):(*S*) = 94.8:5.2, t_R_ = 7.2 min.

(*S*)-1′-Benzyl-4-methoxy-3,4-dihydrospiro[[2]benzopyran-1,4′-piperidine] (**(*S*)-15**)

**(*S*)-13** (50 mg, 0.15 mmol) was dissolved in CH_2_Cl_2_ (5 mL). Trifluoroacetic acid (200 μL) was added and the mixture was stirred for 4.5 h at ambient temperature. Then a 2 M aqueous solution of NaOH was added. After separation of the layers, the aqueous layer was extracted with CH_2_Cl_2_ (3×). The combined organic layers were dried (Na_2_SO_4_), filtered and the solvent was removed *in vacuo*. The residue was dissolved in CH_2_Cl_2_ (10 mL). Benzaldehyde (70 μL, 0.69 mmol) and sodium triacetoxyborohydride (96 mg, 0.45 mmol) were added and the mixture was stirred overnight at ambient temperature. The reaction was stopped by the addition of a 2 M aqueous solution of NaOH. After separation of the layers, the aqueous layer was extracted with CH_2_Cl_2_ (3×). The combined organic layers were dried (Na_2_SO_4_), filtered and the solvent was removed *in vacuo*. The crude product was purified by flash column chromatography twice (1. Ø = 1.5 cm, h = 16 cm, cyclohexane-ethyl acetate = 4:1, V = 5 mL; 2. Ø = 1.5 cm, h = 15 cm, cyclohexane-ethyl acetate = 6:1, V = 5 mL) to give **(*S*)-15** as a yellowish oil (R_f_ = 0.27, cyclohexane-ethyl acetate = 5:5), yield 39 mg (80%). C_21_H_25_NO_2_ (323.4 g/mol). Specific rotation: 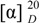: = +7.7 (8.3; CH_2_Cl_2_). Purity (HPLC method 1): 97.3%, t_R_ = 15.6 min. Enantiomeric ratio (HPLC method 2, Daicel Chiralpak IB, 5 μm, 250 mm/4.6 mm, isohexane:methanol = 97:3, flow rate: 1.0 mL/min, injection volume: 5 μL): (*R*):(*S*) = 9.0:91.0, t_R_ = 8.6 min.

Spectroscopic data for **(*R*)-15** and **(*S*)-15**

Exact mass (APCI): *m/z* = 324.1950 (calcd. 324.1958 for C_21_H_26_NO_2_ [M+H]^+^). ^1^H-NMR (CDCl_3_): δ (ppm) = 1.87–1.95 (m, 3H, N(CH_2_C*H*_2_)_2_), 2.13 (td, *J* = 13.0/4.6 Hz, 1H, N(CH_2_C*H*_2_)_2_), 2.36–2.44 (m, 1H, N(C*H*_2_CH_2_)_2_), 2.51 (td, *J* = 13.0/2.5 Hz, 1H, N(C*H*_2_CH_2_)_2_), 2.70–2.79 (m, 2H, N(C*H*_2_CH_2_)_2_), 3.50 (s, 3H, OC*H*_3_), 3.56 (d, *J* = 13.0 Hz, 1H, NC*H*_2_Ph), 3.60 (d, *J* = 13.0 Hz, 1H, NC*H*_2_Ph), 3.93–4.06 (d, *J* = 3.6 Hz, 2H, CHC*H*_2_O), 4.19 (t, *J* = 3.6 Hz, 1H, C*H*CH_2_O), 7.21–7.39 (m, 9H, H_arom._). ^13^C-NMR (CDCl_3_): δ (ppm) = 35.5 (1C, N(CH_2_*C*H_2_)_2_), 37.3 (1C, N(CH_2_*C*H_2_)_2_), 49.4 (1C, N(*C*H_2_CH_2_)_2_), 49.4 (1C, N(*C*H_2_CH_2_)_2_), 56.9 (1C, O*C*H_3_), 61.3 (1C, CH*C*H_2_O), 63.5 (1C, N*C*H_2_Ph), 73.5 (1C, Ar*C*O), 74.1 (1C, *C*HCH_2_O), 125.3 (1C, C_arom._), 126.5 (1C, C_arom._), 127.1 (1C, C_arom._), 128.3 (1C, C_arom._), 128.3 (2C, 3-C_benzyl,_ 5-C_benzyl_), 129.0 (1C, 6-C_arom._), 129.4 (2C, 2-C_benzyl,_ 6-C_benzyl_), 133.1 (1C, 2-C_arom._), 138.7 (1C, 1-Cbenzyl), 142.5 (1C, 1-Carom.). IR (neat): 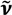
(cm^−1^) = 2924, 2816 (C-H), 1088 (C-O-C), 737 (1,2-disubst. arom.), 737 (monosubst. arom.).

(*R*)-1′-Benzyl-4-ethoxy-3,4-dihydrospiro[[2]benzopyran-1,4′-piperidine] (**(*R*)-16**)

**(*R*)-14** (49 mg, 0.14 mmol) was dissolved in CH_2_Cl_2_ (10 mL). Trifluoroacetic acid (200 μL) was added and the mixture was stirred for 3 h at ambient temperature. Then a 2 M aqueous solution of NaOH (10 mL) was added. After separation of the layers, the aqueous layer was extracted with CH_2_Cl_2_ (3×). The combined organic layers were dried (Na_2_SO_4_), filtered and the solvent was removed *in vacuo*. The residue was dissolved in CH_2_Cl_2_ (10 mL). Benzaldehyde (60 μL, 0.59 mmol) and (after 45 min) sodium triacetoxyborohydride (181 mg, 0.85 mmol) were added and the mixture was stirred overnight at ambient temperature. The reaction was stopped by the addition of a 2 M aqueous solution of NaOH. After separation of the layers, the aqueous layer was extracted with CH_2_Cl_2_ (2×) and ethyl acetate (1×). The combined organic layers were dried (Na_2_SO_4_), filtered and the solvent was removed *in vacuo*. The crude product was purified by flash column chromatography twice (1. Ø = 1.5 cm, h = 17 cm, cyclohexane-ethyl acetate = 4:1, V = 5 mL; 2. Ø = 1.5 cm, h = 17 cm, cyclohexane-ethyl acetate = 6:1, V = 5 mL) to give **(*R*)-16** as a pale yellow oil (R_f_ = 0.31, cyclohexane-ethyl acetate = 5:5), yield 33 mg (70%). C_22_H_27_NO_2_ (337.5 g/mol). Specific rotation: 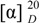: = −3.3 (7.7; CH_2_Cl_2_). Purity (HPLC method 1): 99.1%, t_R_ = 17.0 min.

(*S*)-1′-Benzyl-4-ethoxy-3,4-dihydrospiro[[2]benzopyran-1,4′-piperidine] (**(*S*)-16**)

**(*S*)-14** (63 mg, 0.18 mmol) was dissolved in CH_2_Cl_2_ (5 mL). Trifluoroacetic acid (300 μL) was added and the mixture was stirred for 2 h at ambient temperature. Then a 2 M aqueous solution of NaOH was added. After separation of the layers, the aqueous layer was extracted with ethyl acetate (3×). The combined organic layers were dried (Na_2_SO_4_), filtered and the solvent was removed *in vacuo*. The residue was dissolved in CH_2_Cl_2_ (5 mL). Benzaldehyde (40 μL, 0.39 mmol) and after 15 min, sodium triacetoxyborohydride (120 mg, 0.57 mmol) were added and the mixture was stirred at ambient temperature for 8 h. The reaction was stopped by the addition of a 2 M aqueous solution of NaOH. After separation of the layers, the aqueous layer was extracted with CH_2_Cl_2_ (3×). The combined organic layers were dried (Na_2_SO_4_), filtered and the solvent was removed *in vacuo*. The crude product was purified by flash column chromatography (Ø = 1.25 cm, h = 15 cm, cyclohexane-ethyl acetate = 6:1, V = 5 mL) to give (*S*)-16 as a pale yellow oil (R_f_ = 0.31, cyclohexane-ethyl acetate = 5:5), yield 28 mg (46%). C_22_H_27_NO_2_ (337.5 g/mol). Specific rotation: 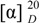: = +2.6 (11.7; CH_2_Cl_2_). Purity (HPLC method 1): 96.6%, t_R_ = 17.0 min.

Spectroscopic data for **(*R*)-16** and **(*S*)-16**

Exact mass (APCI): *m/z* = 338.2130 (calcd. 338.2115 for C_22_H_28_NO_2_ [M+H]^+^) ^1^H-NMR (CDCl_3_): δ (ppm) = 1.20 (t, *J* = 6.9 Hz, 3H, OCH_2_C*H*_3_), 1.74–2.04 (m, 4H, N(CH_2_C*H*_2_)_2_), 2.28–2.24 (m, 2H, N(C*H*_2_CH_2_)_2_), 2.62–2.70 (m, 2H, N(C*H*_2_CH_2_)_2_), 3.50 (s, 2H, NC*H*_2_Ph), 3.58–3.68 (m, 2H, OC*H*_2_CH_3_), 3.79 (dd, *J* = 11.9/5.4 Hz, 1H, CHC*H*_2_O), 3.93 (dd, *J* = 11.9/3.9 Hz, 1H, CHC*H*_2_O), 4.28 (t, *J* = 4.6 Hz, 1H, C*H*CH_2_O), 7.11–7.35 (m, 9H, H_arom._). ^13^C-NMR (CDCl_3_): δ (ppm) = 15.9 (1C, OCH_2_*C*H_3_), 36.3 (1C, N(CH_2_*C*H_2_)_2_), 36.7 (1C, N(CH_2_*C*H_2_)_2_), 49.3 (1C, N(*C*H_2_CH_2_)_2_), 49.5 (1C, N(*C*H_2_CH_2_)_2_), 61.8 (1C, CH*C*H_2_O), 63.6 (1C, N*C*H_2_Ph), 64.8 (1C, O*C*H_2_CH_3_), 72.5 (1C, *C*HCH_2_O), 73.7 (1C, Ar*C*O), 125.2 (1C, C_arom._), 126.6 (1C, C_arom._), 127.1 (1C, C_arom._), 128.0 (1C, C_arom._), 128.2 (1C, C_arom._), 128.3 (2C, C_arom._), 129.4 (2C, C_arom._), 134.2 (1C, C_arom._), 138.7 (1C, C_arom._), 142.4 (1C, C_arom._). IR (neat): 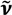
(cm^−1^) = 2928, 2812 (C-H), 1092 (C-O-C ether), 737 (1,2-disubst. arom.), 698 (monosubst. arom_._).

*tert*-Butyl (*R*)-4-(2-ethoxy-2-oxoethoxy)-3,4-dihydrospiro[[2]benzopyran-1,4′-piperidine]-1′-carboxylate (**(R)-17**)

**(*R*)-11** (1.6 g, 5.0 mmol) was dissolved in THF (60 mL). A 1 M solution of lithium bis(trimethyl-silyl)amide (41 mL, 41 mmol) was added and the mixture was stirred for 1 h at ambient temperature. Then ethyl 2-bromoacetate (4.6 mL, 41.5 mmol) and tetrabutylammonium iodide (191 mg, 0.52 mmol) were added and the mixture was heated to reflux overnight. The solvent was removed *in vacuo*. The crude product was purified by flash column chromatography (Ø = 5.5 cm, h = 15 cm, cyclohexane-ethyl acetate = 9:1, V = 65 mL) to give **(*R*)-17** as a pale yellow oil (R_f_ = 0.17, cyclohexane-ethyl acetate = 5:1), yield 1.2 g (59%). C_22_H_31_NO_6_ (405.5 g/mol). Specific rotation: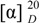: = −14.7 (5.5; CH_2_Cl_2_). Purity (HPLC method 1): 97.3%, t_R_ = 21.3 min.

*tert*-Butyl (*S*)-4-(2-ethoxy-2-oxoethoxy)-3,4-dihydrospiro[2-benzopyran-1,4′-piperidine]-1′-carboxylate (**(*S*)-17**)

**(*S*)-11** (2.0 g, 6.3 mmol) was dissolved in THF (50 mL). A 1 M solution of lithium bis(trimethyl-silyl)amide (50 mL, 50 mmol) was added and the mixture was stirred for 45 min at ambient temperature. Then ethyl 2-bromoacetate (50 mL, 50.5 mmol) and tetrabutylammonium iodide (247 mg, 0.67 mmol) were added and the mixture was heated to reflux overnight. The solvent was removed *in vacuo*. The crude product was purified by flash column chromatography (Ø = 5 cm, h = 17 cm, cyclohexane-ethyl acetate = 9:1, V = 30 mL) to give **(*S*)-17** as a pale yellow oil (R_f_ = 0.17, cyclo-hexane-ethyl acetate = 5:1), yield 1.4 g (55%). C_22_H_31_NO_6_ (405.5 g/mol). Specific rotation: 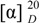: = +14.3 (3.6; CH_2_Cl_2_). Purity (HPLC method 1): 96.0%, t_R_ = 20.9 min.

Spectroscopic data for **(*R*)-17** and **(*S*)-17**

Exact mass (APCI): *m/z* = 406.2224 (calcd. 406.2224 for C_22_H_32_NO_6_ [M+H]^+^). ^1^H-NMR (CDCl_3_): δ (ppm) = 1.29 (t, *J* = 7.1 Hz, 3H, CH_2_C*H*_3_), 1.49 (s, 9H, CO_2_C(C*H*_3_)_3_), 1.68–1.79 (m, 1H, N(CH_2_C*H*_2_)_2_), 1.83–2.02 (m, 3H, N(CH_2_C*H*_2_)_2_), 3.01–3.29 (m, 2H, N(C*H*_2_CH_2_)_2_), 3.93–4.11 (m, 4H, N(C*H*_2_CH_2_)_2_ (2H), CHC*H*_2_O (2H)), 4.17-4.28 (m, 4H, C*H*_2_CH_3_ (2), OC*H*_2_CO_2_ (2)), 4.52 (t, *J* = 3.4 Hz, 1H, C*H*CH_2_O), 7.11 (dd, *J* = 7.6/1.5 Hz, 1H, H_arom._), 7.25–7.35 (m, 2H, H_arom._), 7.55 (dd, *J* = 7.5/1.7 Hz, 1H, H_arom_). ^13^C-NMR (CDCl_3_): δ (ppm) = 14.4 (1C, CH_2_*C*H_3_), 28.7 (3C, CO_2_C(*C*H_3_)_3_), 34.7 (br, 1C, N(CH_2_*C*H_2_)_2_), 37.1 (br, 1C, N(CH_2_*C*H_2_)_2_), 39.5 (br, 1C, N(*C*H_2_CH_2_)_2_), 40.1 (br, 1C, N(*C*H_2_CH_2_)_2_), 61.0 (1C, *C*H_2_CH_3_), 62.0 (1C, CH*C*H_2_O), 65.7 (1C, O*C*H_2_CO_2_), 72.6 (1C, *C*HCH_2_O), 73.6 (1C, CO_2_*C*(CH_3_)_3_), 79.6 (1C, Ar*C*O), 125.0 (1C, C_arom._), 127.0 (1C, C_arom._), 128.8 (1C, C_arom._), 129.5 (1C, C_arom._), 131.8 (1C, C_arom._), 142.1 (1C, C_arom._), 155.0 (1C, *C*O_2_C(CH_3_)_3_), 170.9 (1C, OCH2*C*O2). IR (neat): 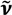
(cm^−1^) = 2974, 2928 (C-H), 1751, 1690 (C=O), 1165, 1099 (C-O-C ether), 759 (1,2-disubst. arom.).

Ethyl (*R*)-2-[(1′-benzyl-3,4-dihydrospiro[[2]benzopyran-1,4′-piperidin]-4-yl)oxy]acetate (**(*R*)-18**)

**(*R*)-17** (86 mg, 0.21 mmol) was dissolved in CH_2_Cl_2_ (4 mL). Trifluoroacetic acid (200 μL) was added and the mixture was stirred overnight at ambient temperature. Then water was added. After separation of the layers, the aqueous layer was extracted with ethyl acetate (3×). The combined organic layers were dried (Na_2_SO_4_), filtered and the solvent was removed *in vacuo*. The residue was dissolved in CH_2_Cl_2_ (2 mL). Benzaldehyde (103 μL, 1.0 mmol) and sodium triacetoxyborohydride (161 mg, 0.76 mmol) were added and the mixture was stirred at ambient temperature for 4 days. The reaction was stopped by the addition of a 2 M aqueous solution of NaOH. After separation of the layers, the aqueous layer was extracted with CH_2_Cl_2_ (3×). The combined organic layers were dried (Na_2_SO_4_), filtered and the solvent was removed *in vacuo*. The crude product was purified by flash column chromatography (Ø = 1.5 cm, h = 14 cm, cyclohexane-ethyl acetate = 3:1, V = 5 mL) to give **(*R*)-18** as a yellowish oil (R_f_ = 0.16, cyclohexane-ethyl acetate = 5:5), yield 36 mg (43%). C_24_H_29_NO_4_ (395.5 g/mol). Specific rotation: 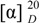: = −16.0 (3.3; CH_2_Cl_2_). Purity (HPLC method 1): 95.4%, t_R_ = 17.7 min.

Ethyl (*S*)-2-[(1′-benzyl-3,4-dihydro-3,4-dihydrospiro[2-benzopyran-1,4′-piperidin]-4-yl)oxy]acetate (**(*S*)-18**)

**(*S*)-17** (1.3 mg, 3.2 mmol) was dissolved in CH_2_Cl_2_ (60 mL). Trifluoroacetic acid (3.5 mL) was added and the mixture was stirred for 7 h at ambient temperature. Then a 2 M aqueous solution of NaOH was added. After separation of the layers, the aqueous layer was extracted with CH_2_Cl_2_ (3×). The combined organic layers were dried (Na_2_SO_4_), filtered and the solvent was removed *in vacuo*. The residue was dissolved in CH_2_Cl_2_ (50 mL). Benzaldehyde (1.0 mL, 9.9 mmol) and, after 15 min, sodium triacetoxyborohydride (2.0 g, 9.4 mmol) were added and the mixture was stirred overnight at ambient temperature. The reaction was stopped by the addition of a 2 M aqueous solution of NaOH and worked up as described for **(*R*)-18**. The crude product was purified by flash column chromatography (Ø = 5 cm, h = 15 cm, cyclohexane-ethyl acetate = 3:1, V = 30 mL) to give **(*S*)-18** as a yellowish oil (R_f_ = 0.16, cyclohexane-ethyl acetate = 5:5), yield 36 mg (43%). C_24_H_29_NO_4_ (395.5 g/mol). Specific rotation: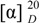: = +15.3 (3.4; CH_2_Cl_2_). Purity (HPLC method 1): 93.1%, t_R_ = 17.3 min.

Spectroscopic data for **(*R*)-18** and **(*S*)-18**

Exact mass (APCI): *m/z* = 396.2177 (calcd. 396.2169 for C_24_H_30_NO_4_ [M+H]^+^). ^1^H-NMR (CDCl_3_): δ (ppm) = 1.30 (t, *J* = 7.1 Hz, 3H, CH_2_C*H*_3_), 1.87–1.95 (m, 3H, N(CH_2_C*H*_2_)_2_), 2.16 (td, *J* = 13.1/4.6 Hz, 1H, N(CH_2_C*H*_2_)_2_), 2.34–2.45 (m, 1H, N(C*H*_2_CH_2_)_2_), 2.47–2.55 (m, 1H, N(C*H*_2_CH_2_)_2_), 2.70–2.81 (m, 2H, N(C*H*_2_CH_2_)_2_), 3.57 (d, *J* = 13.1 Hz, 1H, NC*H*_2_Ph), 3.61 (d, *J* = 13.1 Hz, 1H, NC*H*_2_Ph), 3.98 (dd, *J* = 12.4/3.3 Hz, 1H, CHC*H*_2_O), 4.05 (dd, *J* = 12.4/3.8 Hz, 1H, CHC*H*_2_O), 4.16–4.29 (m, 4H, C*H*_2_CH_3_ (2H), OC*H*_2_CO_2_ (2H)), 4.53 (t, *J* = 3.5 Hz, C*H*CH_2_O), 7.22–7.41 (m, 8H, H_arom._), 7.53–7.57 (m, 1H, H_arom._). ^13^C-NMR (CDCl_3_): δ (ppm) = 14.4 (1C, CH_2_*C*H_3_), 35.1 (1C, N(CH_2_*C*H_2_)_2_), 37.4 (1C, N(CH_2_*C*H_2_)_2_), 49.3 (1C, N(*C*H_2_CH_2_)_2_), 49.4 (1C, N(*C*H_2_CH_2_)_2_), 61.0 (1C, *C*H_2_CH_3_), 61.7 (1C, CH*C*H_2_O), 63.5 (1C, N*C*H_2_Ph), 65.6 (1C, O*C*H_2_CO_2_), 72.7 (1C, *C*HCH_2_O), 73.6 (1C, Ar*C*O), 125.2 (1C, C_arom._), 126.8 (1C, C_arom._), 127.1 (1C, C_arom._), 128.3 (2C, C_arom._), 128.6 (1C, C_arom._), 129.4 (1C, C_arom._), 129.4 (2C, C_arom._), 132.0 (1C, 4a-C_arom._), 138.6 (1C, 1-C_benzyl_), 142.8 (1C, 8a-C_arom._), 170.9 (1C, OCH2*C*O2). IR (neat): 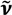
(cm^−1^) = 2920, 2866 (C-H), 1748 (C=O), 1099, 1053 (C-O-C ether), 737 (1,2-disubst. arom.), 698 (monosubst. arom.).

(*R*)-2-[(1′-Benzyl-3,4-dihydrospiro[[2]benzopyran-1,4′-piperidin]-4-yl)oxy]ethanol (**(*R*)-19**)

**(*R*)-18** (480 mg, 1.21 mmol) was dissolved in THF (5 mL). A 1 m solution of LiAlH_4_ in THF (6 mL, 6 mmol) was added and the mixture was stirred overnight at ambient temperature. Then water was added. After separation of the layers, the aqueous layer was extracted with CH_2_Cl_2_ (3×). The combined organic layers were washed with water (2×) and brine (1×), dried (Na_2_SO_4_), filtered and the solvent was removed *in vacuo*. The crude product was purified by flash column chromatography (Ø = 3 cm, h = 15 cm, cyclohexane-ethyl acetate = 5:5, V = 20 mL) to give **(*R*)-19** as a yellowish oil (R_f_ = 0.06, ethyl acetate), yield 254 mg (59%). C_22_H_27_NO_3_ (353.5 g/mol). Specific rotation: 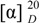: = −2.2 (1.7; CH_2_Cl_2_). Purity (HPLC method 1): 95.1%, t_R_ = 13.8 min.

(*S*)-2-[(1′-Benzyl-3,4-dihydrospiro[[2]benzopyran-1,4′-piperidin]-4-yl)oxy]ethanol (**(*S*)-19**)

**(*S*)-18** (451 mg, 1.14 mmol) was dissolved in THF (10 mL). A 1 M solution of LiAlH_4_ in THF (2.5 mL, 2.5 mmol) was added and the mixture was stirred overnight at ambient temperature. Then water and CH_2_Cl_2_ were added. The reaction was worked up as described for **(*R*)-19**. The crude product was purified by flash column chromatography (Ø = 2 cm, h = 15 cm, cyclohexane-ethyl acetate = 5:5, V = 10 mL) to give **(*S*)-19** as a pale yellow oil (R_f_ = 0.06, ethyl acetate), yield 302 mg (75%). C_22_H_27_NO_3_ (353.5 g/mol). Specific rotation: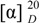: = +2.1 (1.8; CH_2_Cl_2_). Purity (HPLC method 1): 94.3%, t_R_ = 13.9 min.

Spectroscopic data for **(*R*)-19** and **(*S*)-19**

Exact mass (APCI): *m/z* = 354.2039 (calcd. 354.2064 for C_22_H_28_NO_3_ [M+H]^+^). ^1^H-NMR (CDCl_3_): δ (ppm) = 1.85–1.99 (m, 3H, N(CH_2_C*H*_2_)_2_), 2.13 (td, *J* = 13.3/4.6 Hz, 1H, N(CH_2_C*H*_2_)_2_), 2.30–2.44 (s br, 1H, O*H*), 2.40 (td, *J* = 10.9/4.6 Hz, 1H, N(C*H*_2_CH_2_)_2_), 2.45–2.53 (m, 1H, N(C*H*_2_CH_2_)_2_), 2.70–2.79 (m, 2H, N(C*H*_2_CH_2_)_2_), 3.58 (s, 2H, NC*H*_2_Ph), 3.72–3.81 (m, 4H, OC*H*_2_C*H*_2_OH), 3.94 (dd, *J* = 12.3/3.3 Hz, 1H, CHC*H*_2_O), 4.01 (dd, *J* = 12.3/3.9 Hz, 1H, CHC*H*_2_O), 4.36 (t, *J* = 3.6 Hz, 1H, C*H*CH_2_O), 7.22–7.39 (m, 9H, H_arom._). ^13^C-NMR (CDCl_3_): δ (ppm) = 35.4 (1C, N(CH_2_*C*H_2_)_2_), 37.4 (1C, N(CH_2_*C*H_2_)_2_), 49.3 (1C, N(*C*H_2_CH_2_)_2_), 49.4 (1C, N(*C*H_2_CH_2_)_2_), 61.6 (1C, CH*C*H_2_O), 62.2 (1C, OCH_2_*C*H_2_OH), 63.5 (1C, N*C*H_2_Ph), 70.1 (1C, O*C*H_2_CH_2_OH), 73.1 (1C, *C*HCH_2_O), 73.7 (1C, Ar*C*O),125.4 (1C, C_arom._), 126.7 (1C, C_arom._), 127.1 (1C, C_arom._), 128.3 (2C, C_arom._), 128.5 (1C, C_arom._), 128.9 (1C, C_arom._), 129.4 (2C, C_arom._), 133.1 (1C, C_arom._), 138.7 (1C, C_arom._), 142.5 (1C, C_arom._). IR (neat): 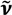
(cm^−1^) = 3399 (O-H), 2924, 2816 (C-H), 1092, 1076 (C-O-C, ether), 741 (1,2-disubst. arom.), 698 (monosubst. arom.).

(*R*)-1′-Benzyl-4-(2-fluoroethoxy)-3,4-dihydrospiro[[2]benzopyran-1,4′-piperidine] (**(*R*)-20**)

(Diethylamino)difluorosulfonium tetrafluoroborate (Xtal-Fluor E^®^, 46 mg, 0.20 mmol) and triethylamine trihydrofluoride (45 μL, 0.28 mmol) were dissolved in CH_2_Cl_2_ (1 mL). The solution was cooled to −78 °C. **(*R*)-19** (48 mg, 0.14 mmol) was added and the mixture was stirred at ‒78 °C for 1 h, then at 0 °C for 1 h and at ambient temperature for 1 h. A 5% aqueous solution of NaHCO_3_ (3 mL) was added and the mixture was stirred for 15 min. The aqueous layer was extracted with CH_2_Cl_2_ (2×). The combined organic layers were dried (Na_2_SO_4_), filtered and the solvent was removed *in vacuo*. The crude product was purified by flash column chromatography (Ø = 0.75 cm, h = 16 cm, cyclohexane-ethyl acetate = 2:1, V = 5 mL) to give **(*R*)-20** as a pale yellow oil (R_f_ = 0.34, cyclohexane-ethyl acetate = 5:5), yield 27 mg (54%). C_22_H_26_FNO_2_ (355.4 g/mol). Specific rotation: 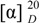: = −6.4 (5.8; CH_2_Cl_2_). Purity (HPLC method 1): 97.0%, t_R_ = 16.4 min.

(*S*)-1′-Benzyl-4-(2-fluoroethoxy)-3,4-dohydrospiro[[2]benzopyran-1,4′-piperidine] (**(*S*)-20**)

(Diethylamino)difluorosulfonium tetrafluoroborate (Xtal-Fluor E^®^, 120 mg, 0.52 mmol) and triethylamine trihydrofluoride (305 μL, 1.87 mmol) were dissolved in CH_2_Cl_2_ (2 mL). The solution was cooled to −78 °C. **(*S*)-19** (120 mg, 0.34 mmol) dissolved in CH_2_Cl_2_ (2 mL), was added and the mixture was stirred at -78 °C for 1 h, then at 0 °C for 1 h and at ambient temperature overnight. A 20% aqueous solution of NaHCO_3_ (4 mL) was added and the mixture was stirred for 15 min. The aqueous layer was extracted with CH_2_Cl_2_ (2×). The combined organic layers were dried (Na_2_SO_4_), filtered and the solvent was removed *in vacuo*. The crude product was purified by flash column chromatography (Ø = 1.5 cm, h = 18 cm, cyclohexane-ethyl acetate = 3:1, V = 5 mL) to give **(*S*)-20** as a pale yellow oil (R_f_ = 0.34, cyclohexane-ethyl acetate = 5:5), yield 86 mg (71%). C_22_H_26_FNO_2_ (355.4 g/mol). Specific rotation: 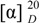: = +5.9 (4.4; CH_2_Cl_2_). Purity (HPLC method 1): 95.1%, t_R_ = 16.1 min.

Spectroscopic data for **(*R*)-20** and **(*S*)-20**

Exact mass (APCI): *m/z* = 356.2032 (calcd. 356.2020 for C_22_H_27_FNO_2_ [M+H]^+^). ^1^H-NMR (CDCl_3_): δ (ppm) = 1.85–2.00 (m, 3H, N(CH_2_C*H*_2_)_2_), 2.10 (td, *J* = 13.3/4.6 Hz, 1H, N(CH_2_C*H*_2_)_2_), 2.39 (td, *J* = 11.6/3.1 Hz, 1H, N(C*H*_2_CH_2_)_2_), 2.48 (td, *J* = 11.6/2.5 Hz, 1H, N(C*H*_2_CH_2_)_2_), 2.69–2.78 (m, 2H, N(C*H*_2_CH_2_)_2_), 3.57 (s, 2H, NC*H*_2_Ph), 3.78–3.87 (m, 1H, OC*H*_2_CH_2_F), 3.87–3.94 (m, 1H, OC*H*_2_CH_2_F), 3.94 (dd, *J* = 12.1/4.7 Hz, 1H, CHC*H*_2_O), 4.01 (dd, *J* = 12.1/3.7 Hz, 1H, CHC*H*_2_O), 4.45 (t, *J* = 4.2 Hz, 1H, C*H*CH_2_O), 4.59 (dt, *J* = 47.7/4.2 Hz, 2H, OCH_2_C*H*_2_F), 7.21–7.38 (m, 8H, H_arom._), 7.41–7.44 (m, 1H, H_arom._). ^13^C-NMR (CDCl_3_): δ (ppm) = 35.7 (1C, N(CH_2_*C*H_2_)_2_), 37.0 (1C, N(CH_2_*C*H_2_)_2_), 49.3 (1C, N(*C*H_2_CH_2_)_2_), 49.4 (1C, N(*C*H_2_CH_2_)_2_), 61.8 (1C, CH*C*H_2_O), 63.5 (1C, N*C*H_2_Ph), 68.0 (d, *J* = 20.3 Hz, 1C, O*C*H_2_CH_2_F), 73.1 (1C, *C*HCH_2_O), 73.6 (1C, Ar*C*O), 83.5 (d, *J* = 179.1 Hz, 1C, OCH_2_*C*H_2_F), 125.3 (1C, C_arom._), 126.7 (1C, C_arom._), 127.2 (1C, C_arom._), 128.4 (2C, C_arom._), 128.7 (1C, C_arom._), 129.5 (2C, C_arom._), 129.6 (1C, C_arom._), 133.0 (1C, C_arom._), 138.4 (1C, C_arom._), 142.5 (1C, Carom.). IR (neat): 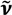
(cm^−1^) = 2934, 2812 (C-H), 1096 (C-O-C, ether), 737 (1,2-disubst. arom.), 698 (monosubst. arom.).

{(*R*)-2-[(1′-Benzyl-3,4-dihydrospiro[[2]benzopyran-1,4′-piperidin]-4-yl)oxy]ethyl} 4-methylbenzene-sulfonate (**(*R*)-21**)

**(*R*)-19** (90 mg, 0.25 mmol) was dissolved in CH_2_Cl_2_ (13 mL). 4-Dimethylaminopyridine (8 mg, 0.07 mmol), triethylamine (176 μL, 1.3 mmol) and 4-toulenesulfonyl chloride (108 mg, 0.57 mmol) were added and the mixture was stirred overnight at ambient temperature. Then a 2 M aqueous solution of NaOH was added. After separation of the layers, the aqueous layer was extracted with CH_2_Cl_2_ (3×). The combined organic layers were dried (Na_2_SO_4_), filtered and the solvent was removed *in vacuo*. The crude product was purified by flash column chromatography (Ø = 1.5 cm, h = 16 cm, cyclohexane-ethyl acetate = 7:3, V = 5 mL) to give **(*R*)-21** as a colorless oil (R_f_ = 0.13, cyclohexane-ethyl acetate = 5:5), yield 59 mg (46%). C_29_H_33_NO_5_S (507.6 g/mol). Specific rotation: 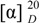: = −5.9 (21.8; CH_2_Cl_2_). Purity (HPLC method 1): 94.5%, t_R_ = 20.3 min.

{(*S*)-2-[(1′-Benzyl-3,4-dihydrospiro[[2]benzopyran-1,4′-piperidin]-4-yl)oxy]ethyl} 4-methylbenzene-sulfonate (**(*S*)-21**)

**(*S*)-19** (140 mg, 0.40 mmol) was dissolved in CH_2_Cl_2_ (20 mL). Triethylamine (274 μL, 2.0 mmol), 4-dimethylaminopyridine (16 mg, 0.13 mmol) and 4-toulenesulfonyl chloride (152 mg, 0.80 mmol) were added and the mixture was stirred overnight at ambient temperature. Then reaction was worked up as described for **(*R*)-21**. The crude product was purified by flash column chromatography (Ø = 2 cm, h = 15 cm, cyclohexane-ethyl acetate = 7:3, V = 10 mL) to give **(*S*)-21** as a yellow oil (R_f_ = 0.13, cyclohexane-ethyl acetate = 5:5), yield 66 mg (32%). C_29_H_33_NO_5_S (507.6 g/mol). Specific rotation: 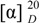 : = +7.5 (23.8; CH_2_Cl_2_). Purity (HPLC method 1): 95.1%, t_R_ = 19.6 min.

Spectroscopic data for **(*R*)-21** and **(*S*)-21**

Exact mass (APCI): *m/z* = 508.2151 (calcd. 508.2152 for C_29_H_35_NO_5_S [M+H]^+^). ^1^H-NMR (CDCl_3_): δ (ppm) = 1.78–1.90 (m, 2H, N(CH_2_C*H*_2_)_2_), 1.91–2.03 (m, 1H, N(CH_2_C*H*_2_)_2_), 2.14 (br t, *J* = 12.9 Hz, 1H, N(CH_2_C*H*_2_)_2_), 2.41 (s, 3H, C*H*_3_), 2.45–2.62 (m, 2H, N(C*H*_2_CH_2_)_2_), 2.77–2.82 (m, 2H, N(C*H*_2_CH_2_)_2_), 3.62 (s, 2H, NC*H*_2_Ph), 3.70–3.87 (m, 3H, OC*H*_2_CH_2_OTos (2H), CHC*H*_2_O (1H)), 3.91 (dd, *J* = 12.2/3.4 Hz, 1H, CHC*H*_2_O), 4.09–4.25 (m, 2H, OCH_2_C*H*_2_OTos), 4.33 (t, *J* = 3.8 Hz, C*H*CH_2_O), 7.08–7.57 (m, 11H, H_arom._), 7.71–7.83 (m, 2H, H-2_tosyl_, H-6_tosyl_). ^13^C-NMR (CDCl_3_): δ (ppm) = 21.8 (1C, *C*H_3_), 35.2 (1C, N(CH_2_*C*H_2_)_2_), 36.8 (1C, N(CH_2_*C*H_2_)_2_), 49.2 (1C, N(*C*H_2_CH_2_)_2_), 49.3 (1C, N(*C*H_2_CH_2_)_2_), 61.7 (1C, CH*C*H_2_O), 63.3 (1C, N*C*H_2_Ph), 66.1 (1C, O*C*H_2_CH_2_OTos), 69.7 (1C, OCH_2_*C*H_2_OTos), 73.0 (1C, *C*HCH_2_O), 73.4 (1C, Ar*C*O), 125.2 (1C, C_arom._), 126.1 (1C, C_arom._), 126.7 (1C, C_arom._), 128.1 (2C, C_arom._), 128.4 (2C, C_arom._), 128.7 (1C, C_arom._), 128.8 (2C, C_arom._), 129.6 (1C, C_arom._), 129,9 (2C, C_arom._), 132.2 (1C, C_arom._), 133.0 (1C, C_arom._), 133.9 (1C, C_arom._), 144.9 (1C, C_arom._), 146.0 (1C, Carom.). IR (neat): 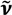
[cm^−1^] = 2924, 2812 (C-H), 1358(m), 1177 (O=S=O), 1096 (C-O-C), 741 (1,2-disubst. arom.), 698 (monosubst. arom.).

### 2.2. Receptor Bindings Studies

#### 2.2.1. Materials

The guinea pig brain and rat liver for the σ_1_ and σ_2_ receptor binding assays were commercially available (Harlan-Winkelmann, Borchen, Germany). Homogenizer: Elvehjem Potter (B. Braun Biotech International, Melsungen, Germany). Cooling centrifuge model Rotina 35R (Hettich, Tuttlingen, Germany) and High-speed cooling centrifuge model Sorvall RC-5C plus (Thermo Fisher Scientific, Langenselbold, Germany). Multiplates: standard 96-well multiplates (Diagonal, Muenster, Germany). Shaker: self-made device with adjustable temperature and tumbling speed (scientific workshop of the institute). Vortexer: Vortex Genie 2 (Thermo Fisher Scientific, Langenselbold, Germany). Harvester: MicroBeta FilterMate-96 Harvester. Filter: Printed Filtermat Typ A and B. Scintillator: Meltilex (Typ A or B) solid state scintillator. Scintillation analyzer: MicroBeta Trilux (all Perkin Elmer LAS, Rodgau-Jügesheim, Germany). Chemicals and reagents were purchased from different commercial sources and of analytical grade.

#### 2.2.2. Preparation of Membrane Homogenates from Guinea Pig Brain

According to [35,36,37]: five guinea pig brains were homogenized with the Potter (500–800 rpm, 10 up-and-down strokes) in 6 volumes of cold 0.32 M sucrose. The suspension was centrifuged at 1,200 × *g* for 10 min at 4 °C. The supernatant was separated and centrifuged at 23,500 × *g* for 20 min at 4 °C. The pellet was resuspended in 5‑6 volumes of buffer (50 mM TRIS, pH 7.4) and centrifuged again at 23,500 × *g* (20 min, 4 °C). This procedure was repeated twice. The final pellet was resuspended in 5‑6 volumes of buffer and frozen (−80 °C) in 1.5 mL portions containing about 1.5 mg protein/mL.

#### 2.2.3. Preparation of Membrane Homogenates from Rat Liver

According to [35,36,37]: two rat livers were cut into small pieces and homogenized with the Potter (500–800 rpm, 10 up-and-down strokes) in 6 volumes of cold 0.32 M sucrose. The suspension was centrifuged at 1,200 × *g* for 10 min at 4 °C. The supernatant was separated and centrifuged at 31,000 × *g* for 20 min at 4 °C. The pellet was resuspended in 5–6 volumes of buffer (50 mM TRIS, pH 8.0) and incubated at room temperature for 30 min. After the incubation, the suspension was centrifuged again at 31,000 × *g* for 20 min at 4 °C. The final pellet was resuspended in 5–6 volumes of buffer and stored at −80 °C in 1.5 mL portions containing about 2 mg protein/mL.

#### 2.2.4. Protein Determination

The protein concentration was determined by the method of Bradford [38], modified by Stoscheck [39]. The Bradford solution was prepared by dissolving 5 mg of Coomassie Brilliant Blue G 250 in 2.5 mL of EtOH (95%, v/v). 10 mL deionized H_2_O and 5 mL phosphoric acid (85%, m/v) were added to this solution, the mixture was stirred and filled to a total volume of 50.0 mL with deionized water. The calibration was carried out using bovine serum albumin as a standard in 9 concentrations (0.1, 0.2, 0.4, 0.6, 0.8, 1.0, 1.5, 2.0 and 4.0 mg/mL). In a 96‑well standard multiplate, 10 µL of the calibration solution or 10 µL of the membrane receptor preparation were mixed with 190 µL of the Bradford solution, respectively. After 5 min, the UV absorption of the protein-dye complex at λ = 595 nm was measured with a platereader (Tecan Genios, Tecan, Crailsheim, Germany).

#### 2.2.5. General Protocol for the Binding Assays

According to [35,36,37]: the test compound solutions were prepared by dissolving approximately 10 µmol (usually 2–4 mg) of test compound in DMSO so that a 10 mM stock solution was obtained. To obtain the required test solutions for the assay, the DMSO stock solution was diluted with the respective assay buffer. The filtermats were presoaked in 0.5% aqueous polyethylenimine solution for 2 h at room temperature before use. All binding experiments were carried out in duplicates in 96-well multiplates. The concentrations given are the final concentrations in the assay. Generally, the assays were performed by addition of 50 µL of the respective assay buffer, 50 µL test compound solution in various concentrations (10^−5^, 10^−6^, 10^−7^, 10^−8^, 10^−9^ and 10^−1^^0^ mol/L), 50 µL of corresponding radioligand solution and 50 µL of the respective receptor preparation into each well of the multiplate (total volume 200 µL). The receptor preparation was always added last. During the incubation, the multiplates were shaken at a speed of 500–600 rpm at the specified temperature. Unless otherwise noted, the assays were terminated after 120 min by rapid filtration using the harvester. During the filtration each well was washed five times with 300 µL of water. Subsequently, the filtermats were dried at 95 °C. The solid scintillator was melted on the dried filtermats at a temperature of 95 °C for 5 min. After solidifying of the scintillator at room temperature, the trapped radioactivity in the filtermats was measured with the scintillation analyzer. Each position on the filtermat corresponding to one well of the multiplate was measured for 5 min with the [^3^H]-counting protocol. The overall counting efficiency was 20%. The IC_50_-values were calculated with the program GraphPad Prism^®^ 3.0 (GraphPad Software, San Diego, CA, USA) by non-linear regression analysis. Subsequently, the IC_50_ values were transformed into K_i_-values using the equation of Cheng and Prusoff [40]. The K_i_-values are given as mean value ± SEM from three independent experiments.

#### 2.2.6. Protocol of the σ_1_ Receptor Binding Assay

According to [35,36,37]: the assay was performed with the radioligand [^3^H]-(+)-pentazocine (22.0 Ci/mmol; Perkin Elmer). The thawed membrane preparation of guinea pig brain cortex (about 100 μg of protein) was incubated with various concentrations of test compounds, 2 nM [^3^H]-(+)-pentazocine, and TRIS buffer (50 mM, pH 7.4) at 37 °C. The non-specific binding was determined with 10 μM unlabeled (+)‑pentazocine. The K_d_-value of (+)-pentazocine is 2.9 nM [41].

#### 2.2.7. Protocol of the σ_2_ Receptor Binding Assay

According to [35,36,37]: the assays were performed with the radioligand [^3^H]DTG (specific activity 50 Ci/mmol; ARC, St. Louis, MO, USA). The thawed membrane preparation of rat liver (about 100 µg of protein) was incubated with various concentrations of the test compound, 3 nM [^3^H]DTG and buffer containing (+)-pentazocine (500 nM (+)-pentazocine in 50 mM TRIS, pH 8.0) at room temperature. The non-specific binding was determined with 10 μM non-labeled DTG. The K_d_ value of [^3^H]DTG is 17.9 nM [42].

### 2.3. Radiochemistry

#### 2.3.1. General

For Solid Phase Extraction (SPE), Sep-Pak^®^ C18 cartridges Plus, Plus short and Plus light (Waters, Eschborn, Germany) as well as Chromabond HR-X^®^ cartridges (Machery-Nagel, Düren, Germany) were tried and C18 cartridges Plus were applied routinely.

Analytical radio-HPLC was performed using an Jasco device series 2000 consisting of autosampler, quaternary pump, degasser, UV-Vis detector, and NaI(Tl)-scintillation detector (bte, Braunschweig, Germany) for gamma detection. A Multospher 120 RP18-AQ column (250x4.6 mm, particle size 5 µm p.s.; CS Chromatographie Service, , Langerwehe, Germany) was applied in gradient mode (0–10 min: 5% MeCN+ 20 mM NH_4_OAc aq.; 10–55 min: 10%–80% MeCN + 20 mM NH_4_OAc aq.) at a flow rate of 1.0 mL/min.

Separation of the crude ^18^F-labeled product was conducted via semi-preparative radio-HPLC in isocratic mode using a Multospher 120 RP18-AQ column (150×10 mm, 5 µm) with 50% acetonitrile + 20 mM NH_4_OAc aq. as eluent at a flow rate of 2 mL/min. The device consisted of an S1021 pump (SYKAM Chromatographie, Fürstenfeldbruck, Germany), UV detector (Well-ChromK-2001, KNAUER, Berlin, Germany), and NaI(Tl)-counter and data acquisition was performed by NINA software version 4.8 rev. 4 (Nuclear Interface, München, Germany).

Radioluminescence thin-layer chromatography (radio-TLC) was performed on alumina coated platelets (Alugram^®^ ALOX N/UV_254_) with petroleum ether/ethyl acetate 7:3 (v/v) as solvent. Radioactive spots were visualised by radioluminescence using a BAS-1800 II system (Bioimaging Analyzer, Fuji Film, Düsseldorf, Germany). Images were evaluated with AIDA 2.31 software (raytest, Straubenhardt, Germany) using the non-radioactive reference compounds after visualization under UV (254 nm).

#### 2.3.2. Synthesis of [^18^F]fluoride Labeled Radiotracer [^18^F]**-(*R*)-20**

Aqueous [^18^F]fluoride was added to a solution of K222 (11.2 mg, 29.7 μmol) and aqueous K_2_CO_3_ (1.78 mg, 12.9 μmol) in acetonitrile (0.5 mL). The solvent was removed azeotropically in an Ar atmosphere under reduced pressure to produce anhydrous reactive K[^18^F]F-K222-carbonate complex. To this mixture a solution of tosylate precursor **(*R*)-21** (2.0–2.5 mg) in acetonitrile (0.5 mL) was added and the reaction mixture was heated to 82 °C for 20 min (total reaction volume 1.0 mL). These optimized reaction conditions led to reproducible labelling yields of 25%–32% (n = 8) as determined by radio-TLC (petroleum ether-ethyl acetate 7: 3 (v/v). TLC retention values: [^18^F]**-(*R*)-20**: R_f_ = 0.83; (*R*)-**21**: R_f_ = 0.27.

### 2.4. *In vitro* stability and lipophilicity of [^18^F]**-(R)-20**

For pharmacological characterization of the radiotracer [^18^F]-*(**R)*****-20**, chemical stability was investigated *in vitro* in different buffer systems over 2 h at 40 °C: 50 mM phosphate buffer (pH 7.2), phosphate-saline-solution (Dulbecco; pH 7.2) and 0.01 M TRIS-HCl (pH 7.4). *In-vitro* stability in native mouse plasma was investigated by incubation of 200 µL plasma plus 5 µL [^18^F]-**(*R*)-20** (~50 MBq) in isotonic NaCl (containing 10% ethanol) for 30 min at 37 °C.

To get information about the blood-brain barrier permeability of [^18^F]-***(R)*-20**, the distribution coefficient logD was determined by conventional shake-flask experiments using *n*-octanol/phosphate buffer pH 7.2, *n*-octanol/phosphate-saline-solution pH 7.2 and *n*-octanol/TRIS-HCl pH 7.4 extraction systems. The amount of the radiotracer [^18^F]-***(R)*-20** in the respective layer was determined using a calibrated γ-counter (Wallac WIZARD, Perkin Elmer, Rodgau-Jügesheim, Germany). The determination of the logD_7,4_ value by HPLC (RP-HPLC; column: ReproSil-Pur AQ, 5 µm, 250 × 4 mm, Dr. Maisch HPLC GmbH, Ammerbruch, Germany; solvent A: NH_4_OAc 20 mM aq., solvent B: acetonitrile using a gradient for B: 0–10 min 10%, 10–40 min 10%–90%; flow: 1 ml/min; detection: 254 nm) was achieved after calibration using reference compounds following the EU guideline 67/548/EWG based on OECD guidelines 2004 [43]. The following reference compounds have been used for calibration (reference compound (*logD*)): phenol (*1.5*), benzene (*2.1*), toluene (*2.7*), benzyl alcohol (*1.1*), *o*-nitrophenol (*1.8*), benzyl chloride (*2.3*), 1,4-dibromobenzene (*3.8*), nitrobenzene (*1.9*), benzophenone (*3.2*), diphenyl ether (*4.2*), fluoranthene (*5.1*), *p*-nitrophenol (*1.9*), chlorobenzene (*2.8*), naphthalene (*3.6*), trichloroethylene (*2.4*), biphenyl (*4.0*), phenanthrene (*4.5*), dibenzyl (*4.8*), thiourea (for determination of t_0_). For the reference compounds as well as **(*R*)-20**, multiple measurements were conducted (n = 3–6). The following regression equation was obtained: log k = 0.07189 × logD + 0.84472 (R^2^ = 0.7988). The logD_7,4_ value of [^18^F]-*(S)*-**20** was recorded six times. Furthermore, the logD value was calculated via ACD ChemSketch2012 software.

### 2.5. Biological Evaluation

#### 2.5.1. General

The experimental protocols were approved by the local ethics committee and conducted according to the national and EU regulations for animal research. Female CD1 mice (10–12 weeks old, 33.8 ± 6.2 g) were obtained from the Medizinisch-Experimentelles Zentrum, Universität Leipzig and housed under a 12 h/12 h light/dark cycle with free access to food and water for at least 24 h before experiments. Animals were sacrificed by CO_2_ asphyxiation after anaesthesia with O_2_/CO_2_ mixture.

#### 2.5.2. *In Vivo* Metabolism of [^18^F]**-(*R*)-20**

The metabolism of [^18^F]-**(*R*)-20** was investigated at 30 min after injection of the radiotracer (166.4 ± 65.4 MBq, dissolved in *ca.* 150 µL saline) into the left or right vena caudalis lateralis. Metabolites were investigated in plasma, urine, brain and liver samples. Urine samples were analyzed directly. Plasma samples were obtained by centrifugation of EDTA blood (12,000 rpm, 4 °C, 10 min) obtained by heart puncture. Brain and liver samples were acquired by homogenization of the organs in ice-cold 50 mM TRIS-HCl buffer (pH 7.4) using a PotterS^®^ Homogeizer (B. Braun). The tissues were treated in a borosilicate glass cylinder by 10 strokes of a PTFE plunge at a speed of 800–1000 min^−1^.

The precipitation of proteins in plasma, brain, and liver samples was performed using twofold extraction of aliquots with ice-cold MeCN (1:4 v/v; for plasma 1:7 v/v), centrifugation of precipitates, and gentle concentration of the combined supernatants (~60°C, argon flow). In addition, precipitation experiments were supplemented by an alternative precipitation method using aqueous MeOH (MeOH/H_2_O 9:1). The percentages of the parent radiotracer and radiometabolites were analysed by radio-HPLC and radio-TLC. The extraction efficiency was controlled using a calibrated γ-counter (Wallac WIZARD, Perkin Elmer).

#### 2.5.3. *Ex Vivo* Autoradiography Studies

The tracer distribution in the brain under control and blocking conditions was determined by *ex vivo* autoradiography studies. [^18^F]-(*R*)-**20** was administered via right vena caudalis lateralis without (31.0 MBq) or with (31.7 MBq) co-application 1 mg/kg haloperidol (Tocris, Bristol, UK) [44]. Animals were sacrificed at 30 min p.i. Blood was collected by heart puncture, the brain quickly removed and transferred on ice, and all samples were weighed. Radioactivity in brain and plasma samples was counted using an automated γ-counter and expressed as percentage of injected dose per gram (%ID/g). For autoradiography, the brain hemispheres were frozen immediately after isolation in 2-methylbutane (Carl Roth, Karlsruhe, Germany) at −30 °C (>2 min). Serial sagittal slices of 12 µm thickness were cut on a cryostat (Microm, Walldorf, Germany) from approx. midline, +500 µm, +1,000 µm and +1,500 µm. Slices were exposed overnight on an imaging plate (SR 2025, Fuji, Tokyo, Japan), scanned afterwards with a high-resolution phosphorimager (HD-CR 35 Bio, raytest). 2D densitometry of the whole brain was performed with AIDA software (raytest). 

## 3. Results and Discussion

### 3.1. Synthesis

The synthesis of enantiomerically pure 2-benzopyrans of type 4 started with 2-bromostyrene (5). Bromine lithium exchange with *n*-BuLi at −78 °C led to an aryllithium intermediate, which was treated with 1-Boc-piperidin-4-one to yield the styrene derivative 6. The Boc-protected piperidone was chosen because the handling (work-up, purification, isolation) of the resulting carbamate was much easier than the handling of alternative benzyl substituted tertiary amines (e.g., 12). Removal of the excess of 1-Boc-piperidin-4-one was performed by LiBH_4_ reduction of the keto group after complete reaction of the ketone with the lithiated vinylbenzene derivative. Flash chromatographic separation of the resulting 1-Boc-piperidin-4-ol resulted in 75% yield of the tertiary alcohol 6.

**Scheme 1:**
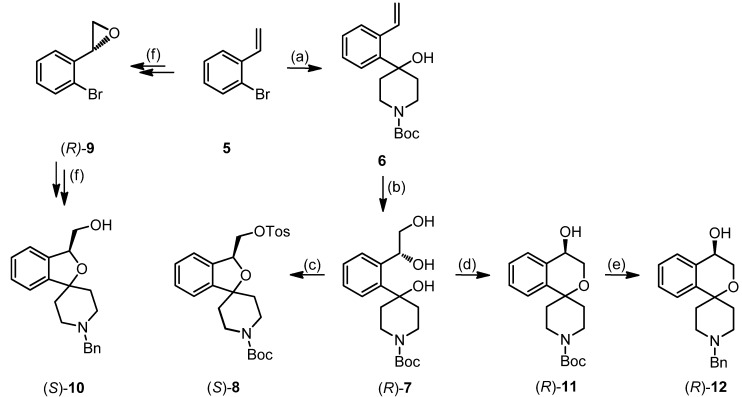
Regioselective synthesis of 2-benzopyrans and 2-benzofurans.

The Sharpless Asymmetric Dihydroxylation of terminal alkene 6 with AD-mix-β employing the standard protocol [45,46,47], led only to low yields of diol (*R)*-7 (Scheme 1). Increasing of the amount of chiral ligand (DHQD)_2_PHAL, oxidant (K_2_OsO_4_) and cooxidant (Na_3_[Fe(CN)_6_] and a longer reaction time did not lead to reproducible high yields of diol (*R*)-7. It was assumed that the reason for the low yields of (*R*)-7 was the low solubility of alkene 6 in a 1:1 *tert*-butanol-water solvent mixture. Despite the addition of different cosolvents (e.g., *tert*-butyl methyl ether, THF) to the solvent mixture, the yields were not improved. However, the yield was considerably raised when increasing the amount of solvent mixture from 10 mL to 60 mL/mmol alkene. The (*R*)-configured triol (*R*)-7 was obtained in a reproducible yield of 82%. Analogously the (*S*)-configured enantiomer (*S*)-7 was accessible in 74% yield, when using AD-mix-α for the dihydroxylation step.

Reaction of triol (*R*)-7 with tosyl chloride, NEt_3_ and DMAP provided the 2-benzofuran (*S*)-8 in 50% yield. It is assumed that tosyl chloride reacts predominantly with the primary OH-moiety of triol (*R*)-7. In the presence of base the resulting primary tosylate forms an oxirane, which is opened by the tertiary alcohol giving the five-membered 2-benzofuran. Finally the primary alcohol reacts with a second equivalent tosyl chloride to form the tosylate (*S*)-8. Reaction of 2-(2-bromophenyl)oxirane (*R*)-9 with n-BuLi and subsequently with 1-Boc-piperidin-4-one led to an analogous 2-benzofuran ((*S*)-10) upon regioselective opening of the oxirane ring by the intermediate lithium alcoholate [35].

*Reagents and conditions*: (a) *n*-BuLi, THF, then 1-Bocpiperidin-4-one, then LiBH_4_, 75%. (b) AD-mix-β, *t*-BuOH/H_2_O = 1:1, 82%. (c) *p*-TosCl, NEt_3_, 4-DMAP, CH_2_Cl_2_, 50%. (d) *p*-TosCl, NEt_3_, Bu_2_SnO, THF, 62%. (e) TFA, CH_2_Cl_2_, then PhCH=O, NaBH(OAc)_3_, CH_2_Cl_2_, 56%. (f) see ref. [33]. Bromostyrene **5** was transformed in the same manner into the corresponding (*S*)-configured enantiomers by using AD-mix-α. (*S*)-**7**: 74%. (S)-**11**: 47%. (S)-**12**: 22%.

However, addition of catalytic amounts of Bu_2_SnO during the tosylation of triol **(*R*)-7** afforded selectively the 2-benzopyran **(*R*)-11**, which was isolated in 62% yield. The addition of Bu_2_SnO was crucial for the synthesis of the 2-benzopyran scaffold. Bu_2_SnO is described as additive for the selective tosylation of the primary OH-moiety of diols by shielding the other OH-moiety [48]. In case of triol **(*R*)-7**, shielding of the secondary OH-moiety by Bu_2_SnO leads to selective activation (*i.e.*, tosylation) of the primary OH-moiety for the nucleophilic substitution.

The enantiomeric purity of (*R*)-11 and (*S*)-11, which was prepared analogously, was analyzed by chiral HPLC using Daicel Chiralpak IB column, resulting in 85% ee for (*R*)-11 and 77.2% ee for (*S*)-11. The moderate enantiomeric excess is explained by the high solvent amount used for the Sharpless Asymmetric Dihydroxylation leading to a lower concentration of the chiral alkaloid ligand due to dilution effect. The lower concentration of the chiral catalyst may lead to an increased amount of uncatalyzed dihydroxylation of alkene 6. This effect has already been described in the literature [33].

For the introduction of the desired benzyl group at the piperidine ring the Boc protective group of (*R*)-11 was cleaved off with trifluoroacetic acid (TFA). Without further purification, the resulting secondary amine was reductively alkylated with benzaldehyde and NaBH(OAc)_3_ to afford the benzyl-substituted alcohol (*R*)-12. After synthesis of the enantiomer (*S*)-12, the enantiomeric purity of both enantiomers was determined by chiral HPLC using a Daicel Chiralpak AD-H column, which resulted in 92.2% ee for (*R*)-12 and 76.2% ee for (*S*)-12.

**Figure 2:**
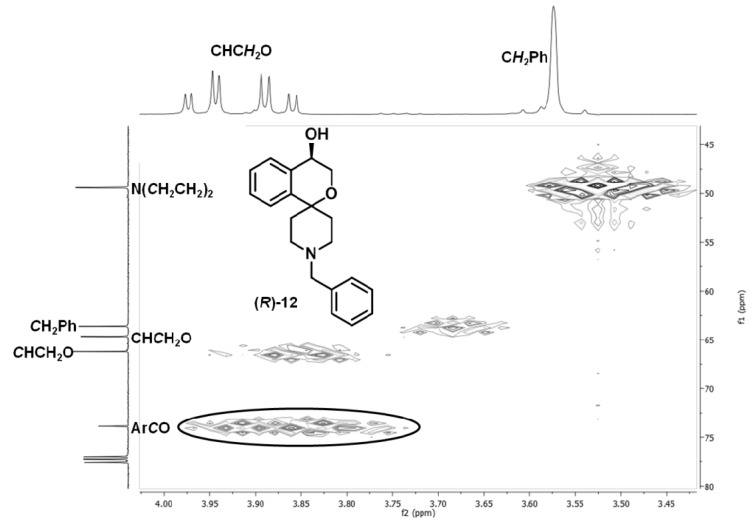
Part of the gHMBC NMR spectrum of 2-benzopyran **(*R*)-12**. The crosspeak between the OCH_2_ protons and the spiro-C-atom is marked.

The structure of the 2-benzopyran **(*R*)-12** was unambiguously identified by comparison of its NMR spectra with those of the hydroxymethyl substituted 2-benzofuran **(*S*)-10**, which was obtained from the oxirane **(*R*)-9** as reported previously by halogen/metal exchange, addition to piperidinone and exchange of the Boc-protective group with a benzyl group [33]. The ^1^H-NMR spectra of the 2-benzopyran **(*R*)-12** and the 2-benzofuran **(*S*)-10** show three doublets of doublets for the ArCH(OR)CH_2_OR substructure. However the chemical shift of the dd for the methine proton of the 2-benzopyran **(*R*)-12** is around 0.8 ppm high-field shifted (4.51 ppm) compared to the dd for the methine proton of the five-membered 2-benzofuran **(*S*)-10** (5.29 ppm). After assigning the ^13^C-NMR signals on the basis of the gHSQC (= gradient heteronuclear single quantum coherence) NMR spectrum, the identity of the 2-benzopyran substructure was proved by 2D gHMBC (= gradient heteronuclear multiple bond correlation) NMR spectroscopy. In this NMR experiment, couplings between protons and carbon atoms over 2–3 bonds are detected. In the 2D gHMBC NMR spectrum of the 2-benzopyran **(*R*)-12** (Figure 2) a coupling between the OCH_2_ signals and the quaternary spiro-C-atom is observed indicating a distance of 2–3 bonds. On the contrary a corresponding crosspeak for the 2-benzofuran **(*S*)-10** is not seen, since four bonds separate the corresponding protons and C-atom.

**Scheme 2:**
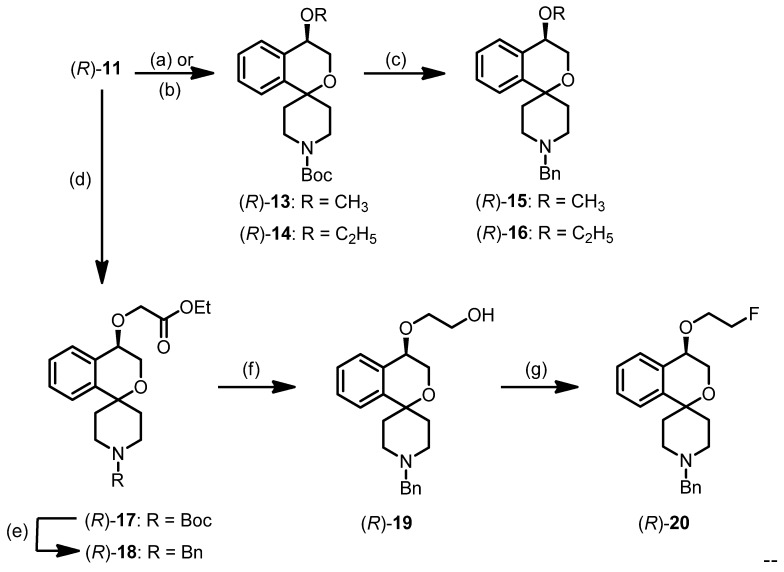
Synthesis of ethers **(*R*)-15**, **(*R*)-16**, and **(*R*)-20**.

Methyl and ethyl ethers **(*R*)-15** and **(*S*)-16** were synthesized by alkylation of the alcohol **(*R*)-11** with methyl iodide and ethyl iodide, respectively, cleavage of the Boc-protective group with trifluoroacetic acid and subsequent reductive alkylation with benzaldehyde and NaBH(OAc)_3_. (Scheme 2) The enantiomeric purity of the methyl ethers was determined via chiral HPLC, resulting in ee-values of 89.6% for **(*R*)-15**, 82% for **(*S*)-15**.

*Reagents and conditions*: (**a**) NaH, THF, then CH_3_I, 99%. (**b**) NaH, THF, then C_2_H_5_I, then LiHMDS, 29%. (**c**) TFA, CH_2_Cl_2_, then benzaldehyde, NaBH(OAc)_3_, CH_2_Cl_2_, 9% (R)-**15**, 70% (R)-**16**. (**d**) LiHMDS, THF, then ethyl 2-bromoacetate, TBAI, 59%. (**e**) TFA, CH_2_Cl_2_, then benzaldehyde, NaBH(OAc)_3_, CH_2_Cl_2_, 43%. (**f**) LiALH_4_, THF, 59%. (**g**) Xtal-Fluor E^®^, NEt_3_ × 3HF, CH_2_Cl_2_, 54%. The (*S*)-configured enantiomers were prepared in the same manner: **(*S*)-13**: 69%. **(*S*)-14**: 50%. **(*S*)-15**: 80%. **(*S*)-16**: 46%. **(*S*)-17**: 55%. **(*S*)-18**: 43%. **(*S*)-19**: 75%. **(*S*)-20**: 71%.

**Scheme 3:**
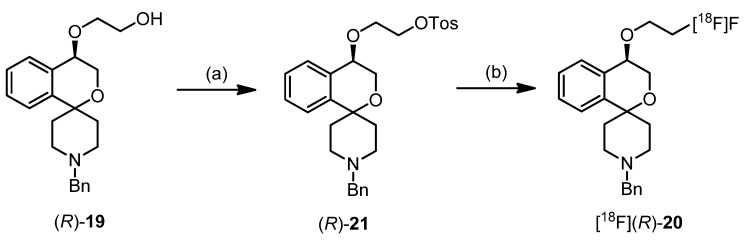
Radiosynthesis of [^18^F]-**(*R*)**-**20**.

In order to introduce a fluorine atom into the substituent in 4-position of the 2-benzopyran ring the alcohol (*S*)-11 was alkylated with ethyl bromoacetate to yield the ester (*R*)-17. Removal of the Boc-protective group and reductive benzylation led to the benzylamine (*R*)-18, which was reduced with LiAlH_4_ to afford the primary alcohol (*R*)-19 in 59% yield. The alcohol (*R*)-19 served as precursor for the introduction of [19F]fluorine as well as the radioactive isotope [^18^F]fluorine into the side chain. Reaction of (*R*)-19 with XtalFluor-E^®^ provided the fluoroethoxy derivative (*R*)-20 in 54% yield. For the radiosynthesis the alcohol (*R*)-11 had to be activated for nucleophilic substitution (see Scheme 3 in the section Radiosynthesis). The corresponding (*S*)-configured enantiomers (*S*)-**17**–**20** were prepared in the same manner.

### 3.2. Receptor Binding Studies

σ_1_ and σ_2_ receptor affinities were measured in competition experiments with radioligands. The σ_1_ receptor binding assay was carried out using a receptor preparation from guinea pig brain and [^3^H]-(+)-pentazocine as a high-affinity and selective radioligand. The σ_2_ receptor affinity was conducted with a receptor preparation from rat liver and [^3^H]di-*o*-toylguanidine ([^3^H]DTG) was used as radioligand. Since DTG is not selective for the σ_2_ subtype over the σ_1_ subtype, σ_1_ receptors binding sites were masked by addition of non-labeled (+)-pentazocine [35,36,37].

The σ_1_ and σ_2_ affinities of the spirocyclic 2-benzopyrans are listed in Table 1. Most of the representatives of this new class of compounds show high σ_1_ affinity with K_i_-values in the low nanomolar range. All 2-benzopyrans are selective towards the σ_2_ receptor subtype with high selectivity factors. With exception of the ethyl ether **16** (eudismic ratio 1), the (*R*) enantiomers represent the eutomers. The eudismic ratio varies from 1.2 (compounds **19**, **20**) indicating low enantioselective receptor binding up to 29 (esters **18**) revealing very high enantioselective receptor interaction.

**Table 1:** σ_1_ and σ_2_ receptor affinities of (*R*)- and (*S*)-configured spirocyclic 2-benzopyrans.

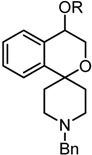						
Entry	Compound	R	Configuration	K_i_ ± SEM [nM]		Selectivity
				σ_1_	σ_2_	σ_1_/σ_2_
1	**(*R*)-11**	H	*R*	5.2 ± 0.3	2150	413
2	**(*S*)-11**	H	*S*	18 ± 5	24% *	>55
3	**(*R*)-15**	Me	*R*	1.2 ± 0.3	1150	958
4	**(*S*)-15**	Me	*S*	8.8 ± 1.1	676	77
5	**(*R*)-16**	Et	*R*	5.0 ± 1.2	954	191
6	**(*S*)-16**	Et	*S*	4.2 ± 0.8	31% *	>235
7	**(*R*)-18**	CH_2_CO_2_Et	*R*	4.0 ± 0.8	552	138
8	**(*S*)-18**	CH_2_CO_2_Et	*S*	114	12% *	>5
9	**(*R*)-19**	CH_2_CH_2_OH	*R*	55 ± 1.0	0% *	>15
10	**(*S*)-19**	CH_2_CH_2_OH	*S*	64 ± 0.9	0% *	>15
11	**(*R*)-20**	CH_2_CH_2_F	*R*	4.7 ± 0.7	14% *	>210
12	**(*S*)-20**	CH_2_CH_2_F	*S*	5.9 ± 2.9	10% *	>170
13	(+)-Pentazocine			5.7 ± 2.2	--	--
14	Haloperidol			6.3 ± 1.6	78 ± 2.3	12

* Inhibition of radioligand binding at a concentration of 1 µM.

As previously reported for other spirocyclic σ_1_ receptor ligands [28], the hydroxy moiety of **(*R*)**-**11** acting as H-bond donor is unfavorable in terms of high σ_1_ receptor affinity (K_i_ = 5.2 nM). Methylation of the OH group led to increased σ_1_ affinity. The methyl ether **(*R*)-15** (K_i_ = 1.2 nM) represents the most potent σ_1_ receptor ligand of this series of spirocyclic 2-benzopyrans. Larger substituents like an ethyl (**(*R*)**-**16**) or an ethoxycarbonylethyl group (**(*R*)**-**18**) reduced the σ_1_ affinity slightly, whereas a substituent with a polar OH group in the side chain resulted in 10-fold reduced σ_1_ affinity (**(*R*)**-**19**: K_i_ = 55 nM).

The fluoroethyl derivatives **20** were synthesized having in mind fluorinated PET tracers for labeling of σ_1_ receptors in the brain. Due to its similar size the fluorine atom is considered as bioisosteric replacement of a proton, but due its high electronegativity it is also regarded as a bioisostere of an OH moiety. As summarized in Table 1 the ethyl derivative **(*R*)-16** and the fluoroethyl derivative **(*R*)-20** show very similar σ_1_ receptor affinity proving the H/F bioisosterism. On the contrary the fluoroethyl derivative (R)-**20** is 10-fold more active than the hydroxyethyl derivative **(*R*)-19** indicating that in this compound class the hydroxy moiety and the fluorine atom cannot be exchanged bioisosterically by each other. The very high σ_1_ affinity of the (*R*)-configured fluoroethyl derivative **(*R*)-20** (K_i_ = 4.7 nM), which is slightly higher than the σ_1_ affinity of the (*S*)-configured enantiomer **(*S*)-20**, rendered this compound a promising candidate for molecular imaging of σ_1_ receptors after labeling with [^18^F]fluorine.

### 3.3. Radiosynthesis

For the radiosynthesis of [^18^F]-**(*R*)-20** (K_i_ = 4.7 nM) the alcohol **(*R*)-19** was converted into the tosylate precursor **(*R*)-21** upon treatment with *p*-TosCl. (Scheme 3)

*Reagents and Conditions*: (**a**) p-TosCl, NEt_3_, 4-DMAP, CH_2_Cl_2_, RT, overnight, 46%; **(*S*)-21** was obtained in 32% yield starting with **(*S*)-19**. (**b**) K[^18^F]F, Kryptofix K222, acetonitrile, 82 °C, 20 min.

**Figure 3:**
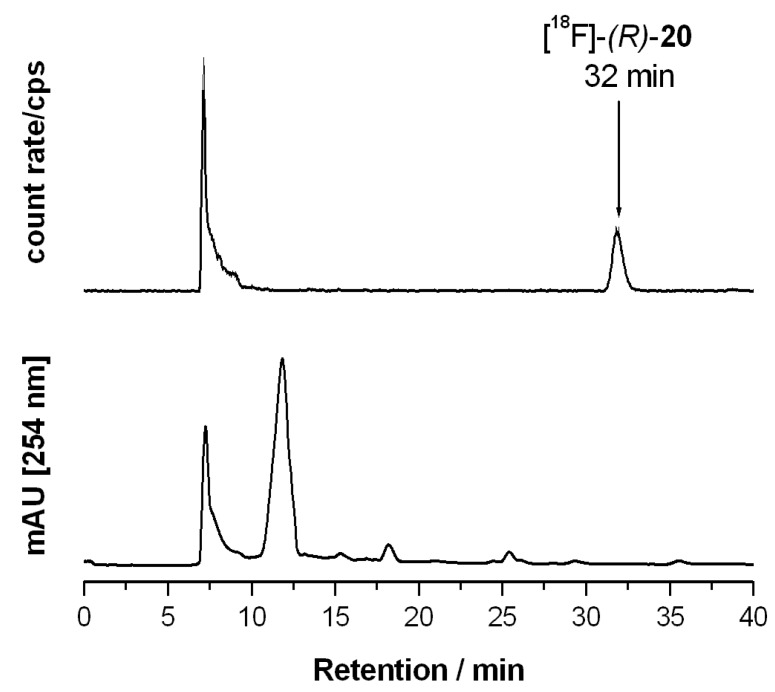
Semi-preparative HPLC separation of the crude labelling mixture of [^18^F]-**(*R*)-20**. HPLC chromatogram with radioactivity detection (above) and UV detection (below). The identity of the final radioactive product, [^18^F]-**(*R*)-20**, was validated with stable [19F]-**(*R*)**-**20** as reference.

The one-step introduction of ^18^F was performed by a S_N_2 substitution of the precursor (*R*)-**21** with [^18^F]fluoride using the K[^18^F]F-K222-carbonate complex, prepared from a 1:1 mixture of K_2_CO_3_ and Kryptofix K222. Using this complex, the precursor (*R*)-**21** was readily transformed into the ^18^F-labeled radiotracer by heating in acetonitrile at 82 °C for 20 min. According to radio-TLC and radio-HPLC analyses, only a few radioactive by-products were formed. Thus, the crude reaction mixture was diluted with water to 4 mL and directly applied to semi-preparative HPLC. The radiotracer eluted at ca. 32 min and was completely free from radioactive and non-radioactive impurities. (Figure 3) Interestingly, a considerable part of ^18^F activity, mainly from highly polar components remained in the stainless steel loop. Using a PEEK loop in the HPLC device resulted in about 80%–90% elution of [^18^F]-(*R*)-20. Combined isolated fractions were diluted with water (50 mL), adsorbed on a Sep-Pak C18 Plus cartridge and desorbed with pure MeOH in small portions. Adsorption of the total activity (≥95%) and elution with MeOH (≥95%) were achieved resulting in a total volume of 1.25 to 1.5 mL. The solvent was carefully evaporated at 60 °C, and the final product was dissolved in 0.9% NaCl solution containing 5% EtOH.

[^18^F]-**(*R*)**-**20** was obtained with radiochemical yields of 18%ߝ20%, radiochemical purity of ≥97%, and specific activities of about 175ߝ300 GBq/µmol within a total synthesis time of about 2 h.

### 3.4. *In Vitro* Stability and Lipophilicity of *[^18^F]***-(R)-20**

According to radio-TLC the radiotracer [^18^F]-**(*R*)-20** proved to be chemically stable in phosphate buffer, Dulbecco buffer and in native mouse plasma over 30 min at 37 °C. No defluorination was observed.

Determination of the distribution coefficient by the shake flask method provided a logD_7.4_ value of 1.78 ± 0.8. This value is in good accordance with the calculated logD_7.4_ value of 1.68 (ACD ChemSketch 2012). The logD_7.4_ value obtained by HPLC method, however, differed by one order of magnitude (2.97±0.32) from these values. The range of the experimentally determined logD_7.4_ values is very similar to the corresponding logD_7.4_ values of fluspidine (**2b**, 1.5–1.8), which has been shown to have excellent brain uptake properties.

### 3.5. Biological Evaluation

#### 3.5.1. Metabolic Stability of [^18^F]**-(*R*)-20** in Mice

**Figure 4:**
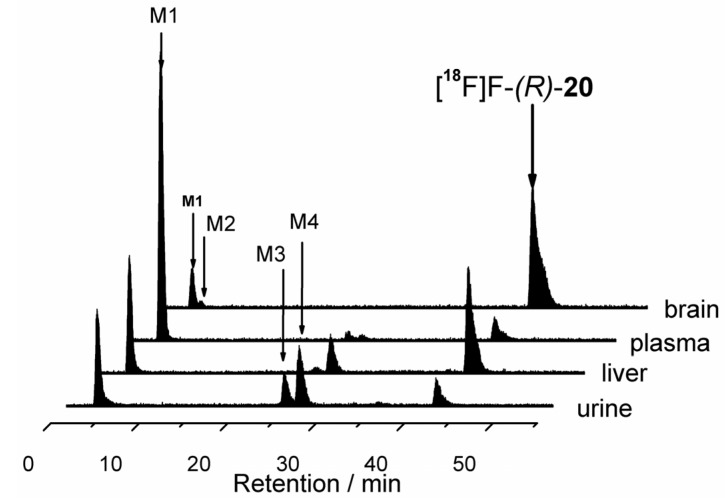
Stacked analytical radio-HPLC profiles of brain, plasma, liver and urine samples; samples were collected 30 min after injection of the radiotracer [^18^F]-**(*R*)-20**; t_R_ of the parent compound *ca.* 42 min.

The existence of radiometabolites after injection of the radiotracer [^18^F]-**(*R*)-20** in mice was analyzed by radio-HPLC and radio-TLC analyses. In Figure 4 HPLC chromatograms of brain, plasma, liver and urine samples are presented in a combined manner.

In brain samples (n = 4) the fraction of the non-metabolized radiotracer accounted for 91%–95% (radio-TLC) and 89%–92% (radio-HPLC) with good reproducibility. Only one highly polar (hydrophilic) radiometabolite (M1) was detected. Its retention time (*t*_R_ 3.6 min) was similar to but not identical with the retention time of [^18^F]fluoride. 

Analysis of the plasma samples (n = 3) at 30 min p.i. revealed fast biotransformation (15% and 12% of [^18^F]-**(*R*)**-**20** remained unchanged as determined by acetonitrile and methanol extraction). Results from radio-HPLC and radio-TLC agreed well. The recovery of total radioactivity was 75% for acetonitrile extraction and up to 90% for methanol extraction. The main radiometabolite in plasma samples was the very polar metabolite M1 (80%–90% of total radioactivity at 30 min p.i.). Additionally, two small peaks for metabolites M2 (t_R_ ~24.7 min) and M3 t_R_ ~26.0 min) were detected with an intensity of lower than 3%.

In urine samples (n = 3), a large amount of radiometabolites and a very low amount of the parent radiotracer [^18^F]-**(*R*)-20** (non-metabolized radiotracer accounting for 2%–20% of total radioactivity) was observed.

The results obtained by analysis of liver homogenates are based on a single experiment and should be treated with caution. About 50% to 56% of the parent radiotracer [^18^F]-**(*R*)-20** (determined after acetonitrile and methanol extraction, respectively) remained unchanged after 30 min. The recovery of radioactivity was 45% for acetonitrile and 89% for methanol extraction. The same radiometabolite profile as in plasma and urine samples was found.

#### 3.5.2. Comparison of Biotransformation of [^18^F]**-(*R*)-20** with Established PET Tracers [^18^F]**2b** and [^18^F]**2c**

With respect to biotransformation the new 2-benzopyran based radiotracer [^18^F]-**(*R*)**-**20** behaved quite different, when compared with the ^18^F labelled radiotracers [^18^F]**2b** and [^18^F]**2c** with a 2-fluoroethyl or 3-fluoropropyl side chain. After application of all three fluorinated radiotracers radioactive metabolites were not found in the brain. However, in the plasma only 15% of the unchanged tracer [^18^F]-**(*R*)-20** was detected, whereas 89% and 86% of the unchanged parent compounds [^18^F]**2b** and [^18^F]**2c** were found in the plasma. Also the amount of parent compound [^18^F]-**(*R*)**-**20** in liver samples was lower (50%) compared with those of [^18^F]**2b** (69%) and [^18^F]**2c** (65%) in liver samples. These results indicate that the 2-benzopyran based radiotracer [^18^F]-**(*R*)**-**20** with a 2-fluoroethoxy side chain underwent faster biotransformation than the analogous 2-benzofuran based tracers [^18^F]**2b** and [^18^F]**2c** with a fluoroethyl or fluoropropyl side chain. The faster metabolic degradation of [^18^F]-**(*R*)**-**20** could be due to the additional ether in the side chain [49,50]. Nevertheless, as no brain-permeable radiometabolites of the 2-benzopyran-based [^18^F]-**(*R*)**-**20** were detected, this radiotracer is applicable for brain imaging studies by PET. 

#### 3.5.3. *Ex Vivo* Autoradiography Studies

In order to investigate the spatial distribution of [^18^F]-**(*R*)-20** and the specificity of its uptake in the mouse brain, *ex vivo* autoradiography studies were performed under control and blocking conditions. Haloperidol co-application reduced the uptake of radioactivity in the brain at 30 min p.i. by 34% (3.40% ID/g *vs.* 2.24% ID/g under control and blocking conditions, respectively). Although this result indicates target specific binding of [^18^F]-**(*R*)**-**20**, the corresponding total-to-nonspecific binding ratio of [^18^F]-**(*R*)**-**20** of 1.5 at 30 min p.i. is lower than previously reported ratios of approx. 3 at 30 min after injection of [^18^F]fluspidine (**2b**) [29] or the corresponding ^18^F-labeled fluorobutyl-radiotracer **2d** [29]. This may be attributed to the lower σ_1_ affinity of [^18^F]-**(*R*)-20** (*K*_i_ = 4.7 nM) compared to fluspidine (**2b**) (*K*_i_ = 0.59 nM) and the fluorobutyl derivative **2d** (*K*_i_ = 1.2 nM).

**Figure 5:**
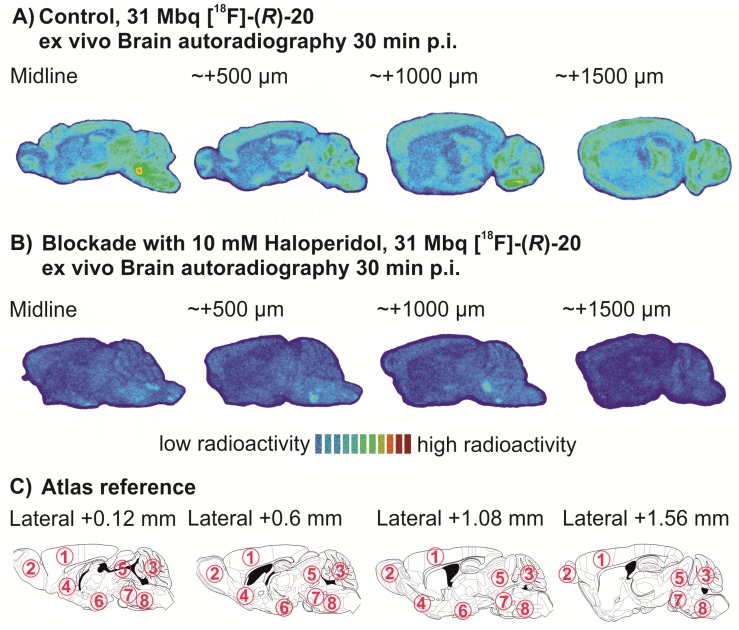
*Ex vivo* brain autoradiographs under control (**A**) and blockade (**B**) conditions. Anatomical reference (Mouse Atlas, Paxinos & Franklin) is shown in C. Numbers indicate the following major brain regions: 1, Cerebral cortex; 2, Olfactory bulb; 3, Cerebellum; 4, Basal forebrain; 5, Midbrain; 6, Hypothalamus; 7, Pons; 8, Brainstem.

Binding of the radiotracer [^18^F]-**(*R*)**-**20** in the mouse brain, shown in Figure 5A, corresponds to binding of [^18^F]fluspidine [29] and [^3^H]1,3-di(2-tolyl)guanidine [7]. Under control conditions, the highest uptake of [^18^F]-**(*R*)**-**20** was detected in the whole brainstem (most prominent in the facial nucleus) and the pons (pontine reticular nucleus). High to moderate uptake was noted in midbrain, cortex, hippocampus, cerebellum and layers of the olfactory bulb. Low uptake was found in parts of the olfactory bulb, striatum and basal forebrain. 2D densitometric evaluation of whole brain sections confirmed the general blocking effect of haloperidol with a mean decrease of radiotracer uptake in the slices of the whole brain hemisphere of about 40%. (Figure 5B) This effect corresponds to the 34% decrease of uptake reflected by the %ID/g values reported above.

Brain autoradiographs also revealed that regions with high radioligand binding (e.g., pontine nuclei, facial nucleus) showed residual binding (Figure 5B) after co-administration of haloperidol.

## 4. Conclusions

In this manuscript the asymmetric synthesis of spirocyclic 2-benzopyrans in enantiomerically pure form is described for the first time. The key step of the synthesis is the asymmetric dihydroxylation according to Sharpless, which allows the preparation of 4-substituted spirocyclic 2-benzopyrans. These compounds represent a new type of potent and subtype selective σ_1_ receptor ligands. Some of the compounds show an eudismic ratio up to 29 (ester 18), indicating an enantioselective interaction of the σ_1_ receptor with these ligands. The very potent fluorinated fluoroethoxy derivative **(*R*)-20** was developed as fluorinated PET-tracer. The radiosynthesis based on a one-step nucleophilic substitution of tosylate **(*R*)-21** provided the PET tracer [^18^F]-**(*R*)-20** in 18%–20% radiochemical yield. Whereas radiometabolites of [^18^F]-**(*R*)-20** were not found in the brain, plasma, liver and urine samples showed a large amount of radiometabolites. Obviously the 2-benzopyran derivative [^18^F]-**(*R*)-20** with a fluoroethoxy side chain is faster metabolized than the corresponding benzofuran-based radiotracers [^18^F]**2b** and [^18^F]**2c** with fluoroethyl or fluoropropyl side chains. In *ex vivo* autoradiography experiments brain regions with high σ_1_ receptor expression are labeled selectively. Altogether, [^18^F]-**(*R*)-20** represents a very good alternative fluorinated PET tracer for imaging of σ_1_ receptors in the brain.
